# Promoting Treg Polarization‐Mediated Anti‐Scar and Appendage Regeneration in Wound Healing

**DOI:** 10.1002/advs.202510815

**Published:** 2026-02-24

**Authors:** Yiwen Yang, Mingjie Kuang, Jiacheng Shi, Ruiyan Li, Yuhan Zhang, Xinting Liu, Runtan Li, Yong Kang, Bin Yao, Shuquan Zhang, Xiaoyuan Ji

**Affiliations:** ^1^ Academy of Medical Engineering and Translational Medicine Medical College Tianjin University Tianjin China; ^2^ Department of Orthopedics Shandong Provincial Hospital Affiliated with Shandong First Medical University Jinan Shandong China; ^3^ State Key Laboratory of Advanced Medical Materials and Devices Tianjin University Tianjin China; ^4^ Integrated Chinese and Western Medicine Hospital Tianjin University Tianjin China; ^5^ Department of Gastric Surgery Tianjin Medical University Cancer Institute & Hospital National Clinical Research Center for Cancer Tianjin Key Laboratory of Digestive Cancer Tianjin's Clinical Research Center for Cancer Tianjin China

**Keywords:** antiscar regeneration, L‐lactic acid, piezoelectric stimulation, regulatory T cells, wound healing

## Abstract

Scar formation and the loss of appendages are major challenges in skin repair following injury. To address these issues, this study introduces a novel bilayer scaffold composed of poly(lactic acid) (PLA) and gelatin methacryloyl (GelMA), which is designed to enhance wound healing and reduce scar formation. Owing to its piezoelectric properties, the upper PLA layer generates an electric field that promotes cell migration, providing dynamic stimulation to the healing tissue. Moreover, the GelMA lower layer functions as an effective drug delivery platform, releasing L‐lactic acid (LA) and bone morphogenetic protein 4 (BMP4). LA plays a crucial role in inducing regulatory T‐cell (Treg) polarization and modulating the macrophage phenotype, thereby fostering an anti‐inflammatory and antifibrotic immune environment. BMP4, on the other hand, inhibits the differentiation of fibroblasts (FBs) into myofibroblasts (MFBs), a key process in excessive collagen deposition, thus preventing fibrosis. In vitro, the bilayer scaffold enhances Treg polarization, promotes the M2 macrophage phenotype, and stimulates FB activity, contributing to a healthier wound healing environment. In vivo, the scaffold accelerated wound closure and promoted tissue regeneration, significantly reducing scar formation. This innovative dual‐layered scaffold demonstrates great promise in advancing wound healing by promoting scarless tissue regeneration, offering a potential therapeutic strategy for chronic wound treatment and scar prevention. The integration of piezoelectric stimulation with controlled drug release provides a unique and multifaceted approach, making this strategy a highly innovative and clinically relevant solution for improving wound healing outcomes.

## Graphical abstract

This study develops a PLGA@LA‐BMP4‐PG bilayer scaffold to address scar formation and appendage loss in skin repair. The piezoelectric PLA layer enhances cell migration via electric fields, while GelMA delivers LA promoting Tregs polarization and BMP4 inhibiting FBs differentiation. In vitro and in vivo tests demonstrate appendages regeneration and scarless healing, offering clinical potential for wound therapy.

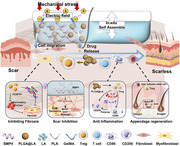

## 1 Introduction

Scar formation is an intrinsic and inevitable outcome of the wound healing process, functioning as a reparative mechanism to restore tissue integrity following injury. Wound repair progresses through three phases: inflammation, proliferation, and remodeling. In the inflammatory phase, platelet aggregation initiates clot formation, whereas immune cells, particularly macrophages, clear debris and release proinflammatory cytokines (IL‐1, TNF‐α), activating fibroblasts (FBs). The proliferative phase is characterized by FB proliferation, extracellular matrix (ECM) secretion, and angiogenesis to sustain tissue regeneration. However, excessive FB activation leads to the overproduction of collagen, a key factor in fibrosis and scar formation. The remodeling phase reorganizes collagen fibers, but in scars, disorganized ECM results in mechanical weakness and loss of functional skin structures [1]. Persistent FB activation and disrupted immune regulation contribute to pathological scarring, such as hypertrophic scars and keloids. Understanding these mechanisms is essential for developing targeted strategies to modulate immune responses and FB activity, promoting regenerative healing while minimizing fibrosis [2].

Several antiscar strategies, including fibrosis inhibitors and tissue engineering approaches, have been developed. Fibrosis inhibitors, such as transforming growth factor‐β (TGF‐β) inhibitors, aim to reduce collagen deposition and ECM accumulation by targeting key signaling pathways involved in the fibrotic process. However, these inhibitors may delay wound closure and carry the risk of incomplete healing. On the other hand, tissue engineering strategies involve incorporating skin‐derived cells or stem cells into biomaterials to promote tissue regeneration. While tissue engineering methods show promise, they face significant challenges in terms of production complexity and clinical translation. Other approaches include the use of growth factors, mechanical force modulation, and gene therapy to regulate the healing environment, although these methods also have limitations, such as unpredictable outcomes or high costs.

The role of the microenvironment in disease progression, including scar formation, is gaining increasing recognition. While considerable research has focused on the cellular and molecular mechanisms of wound healing, less attention has been given to actively regulating the wound microenvironment to prevent pathological scarring. Scar formation is a dynamic process influenced by interactions between immune cells, FBs, extracellular matrix (ECM) components, and cytokines. Maintaining a balanced immune response is essential during wound healing, as excessive inflammation or FB activation can lead to excessive ECM deposition and fibrosis.

Regulatory T cells (Tregs) play a pivotal role in modulating the immune environment during wound healing. They exert anti‐inflammatory effects by suppressing proinflammatory cells and promoting the secretion of anti‐inflammatory cytokines, indirectly facilitating wound healing [3, 4]. Tregs help regulate inflammation within an optimal range, preventing excessive immune responses and fibrosis. Studies have shown that Tregs in the skin can modulate FB activation, with the transcription factor GATA3 playing a role in inhibiting FB activation and dermal fibrosis [5]. The core mechanism by which GATA3 suppresses fibrosis lies in its role as a master transcription factor that confers a unique identity and functional specificity upon skin‐resident Tregs. This enables them to precisely maintain cutaneous immune homeostasis, thereby indirectly yet effectively preventing the onset of fibrosis. This process is initiated by the specific high expression of GATA3 within skin Tregs, which is not a stochastic event but rather a functional adaptation to the local skin microenvironment often challenged by insults that predispose individuals toward a profibrotic type 2 immune response. The programmed expression of GATA3 drives a transcriptomic shift in skin Tregs, essentially equipping them at the genetic level with the molecular tools necessary to recognize, respond to, and suppress profibrotic immune activities. Leveraging this unique identity, skin Tregs precisely target their suppressive function toward specific immune cell subsets that drive fibrosis. Through direct cell‒to‐cell contact and the secretion of specific inhibitory molecules, they actively suppress the activation, proliferation, and effector functions of these target immune cells. This precise “upstream interception” is critical, as it involves the key aberrant activation signals relayed from the immune system to tissue‐repair cells (FBs). Under physiological conditions, moderate activation aids wound healing; however, in the absence of Treg‐mediated restraint, persistent strong signaling drives the excessive activation and differentiation of FBs into myofibroblasts (MFBs). The function of GATA3^+^ Tregs is to prevent this very transformation, thereby averting the massive generation and prolonged persistence of MFBs, the primary executors of fibrosis. In the context of sustained aberrant signaling, MFBs are prevented from engaging in the uncontrolled synthesis and secretion of extracellular matrix components. This ultimately prevents the pathological accumulation of collagen and other proteins, as well as destructive thickening of the dermis, thereby preserving the normal architecture and function of the skin tissue. Moreover, Tregs can reduce fibrosis through cytokines such as IL‐4 and IL‐13 or through direct interactions with FBs [6]. Tregs are also involved in regenerating skin appendages, such as hair follicles and sweat glands, which are essential for restoring functional tissue rather than just for aesthetic healing [7, 8, 9, 10]. Despite these promising findings, Tregs have not been extensively studied for their potential in antiscarring therapies, and effectively regulating Tregs at the wound site remains a significant challenge. Our previous research demonstrated that Tregs are highly expressed in tumor microenvironments due to glycolysis‐induced L‐lactic acid (LA) accumulation, which promotes Treg polarization [11]. This discovery highlights the potential of manipulating Tregs to create a favorable immune environment for scar prevention, opening new avenues for targeted therapies in wound healing.

**FIGURE 1:**
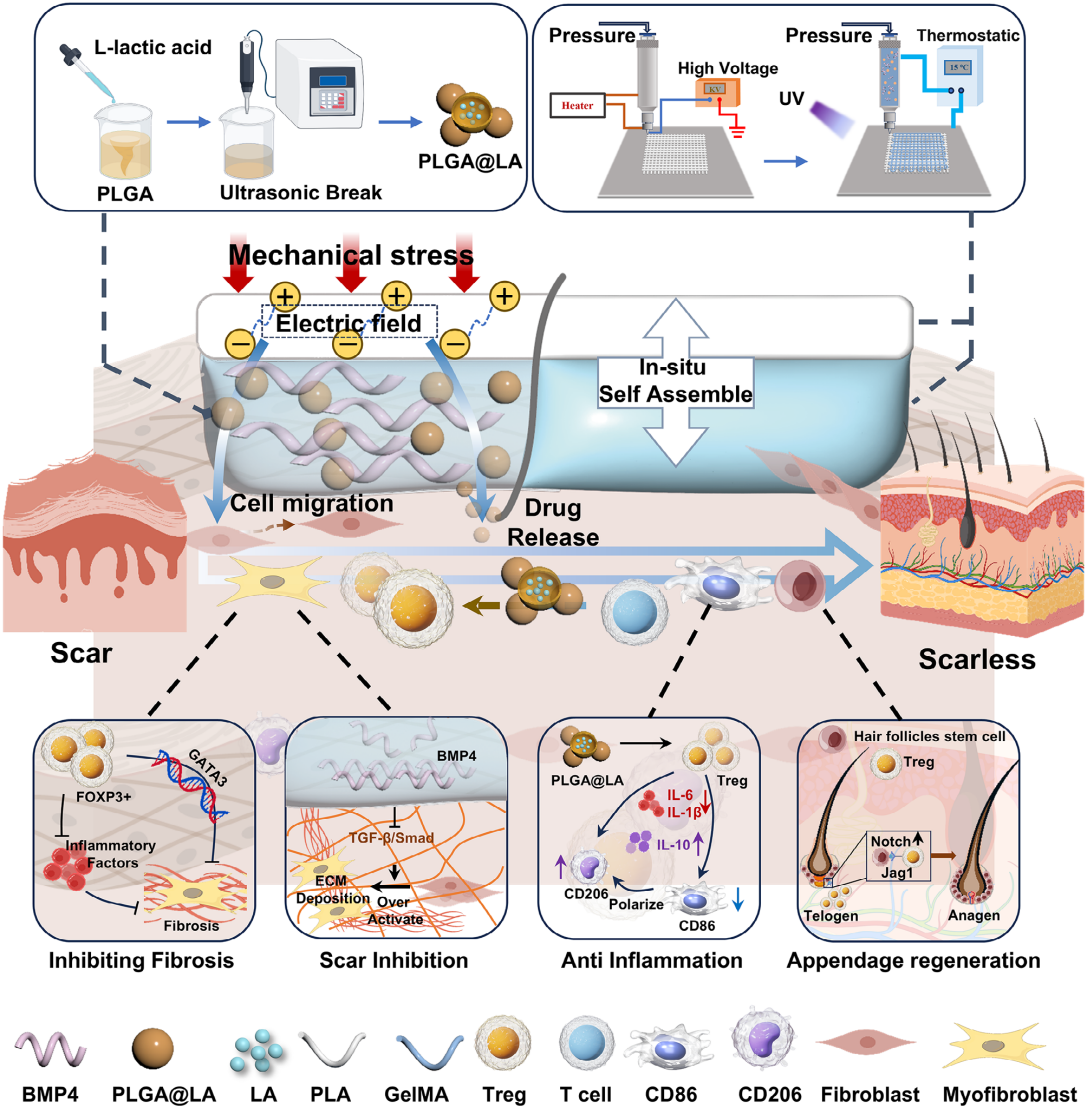
Schematic diagram of the preparation and in vivo application of a bilayer scaffold for inducing scarless wound healing. The PLGA@LA‐BMP4‐PG bilayer scaffold consists of an upper layer of the PLA scaffold and a lower layer of the photocross‐linked GelMA hydrogel containing PLGA@LA nanoparticles and BMP4. During the wound healing process, mechanical stress induced a piezoelectric effect in the PLA scaffold, and the resulting electric field promoted cell migration and drug release from the lower hydrogel. The PLGA@LA nanoparticles released from the lower hydrogel can promote Treg polarization, which can reduce the secretion of inflammatory factors and attenuate fibrosis, in addition to promoting the regeneration of skin appendages. The BMP4 protein can effectively prevent the overactivation of FBs into MFBs and inhibit scarring.

This study proposes a novel biomaterial‐based strategy that integrates immune modulation and fibrosis inhibition to improve wound healing and reduce scar formation. Despite advances in antiscarring therapies, a significant knowledge gap remains in achieving synchronized regulation of the immune microenvironment and FB activity during tissue repair. To address this, we developed a bilayer scaffold that concurrently delivers LA to promote Treg‐mediated immune tolerance and bone morphogenetic protein 4 (BMP4) to suppress MFB activation and fibrosis [12]. This dual‐function therapeutic approach involves delivery via a bilayer scaffold, which is fabricated in situ via 3D printing technology. The upper layer comprises a poly(lactic acid) (PLA) scaffold with piezoelectric properties designed to generate electrical stimulation that enhances cellular migration and proliferation. The lower layer, composed of gelatin methacryloyl (GelMA), serves as a bioactive matrix for drug delivery, promoting localized and sustained release of LA and BMP4. The mesh‐like architecture of both layers facilitates cell adhesion, proliferation, and vascularization while alleviating wound hypoxia, further enhancing regenerative potential. By integrating immune regulation, fibrosis suppression, and advanced scaffold engineering, this study introduces a multifunctional therapeutic platform that addresses the limitations of current wound healing strategies (Figure 1). The ability to precisely modulate immune responses and FB activity within the wound microenvironment provides a promising avenue for achieving high‐quality, functional tissue regeneration with minimal scarring.

## 2 Results

### 2.1 Inhibition of Fibrosis by Tregs and BMP4

**FIGURE 2:**
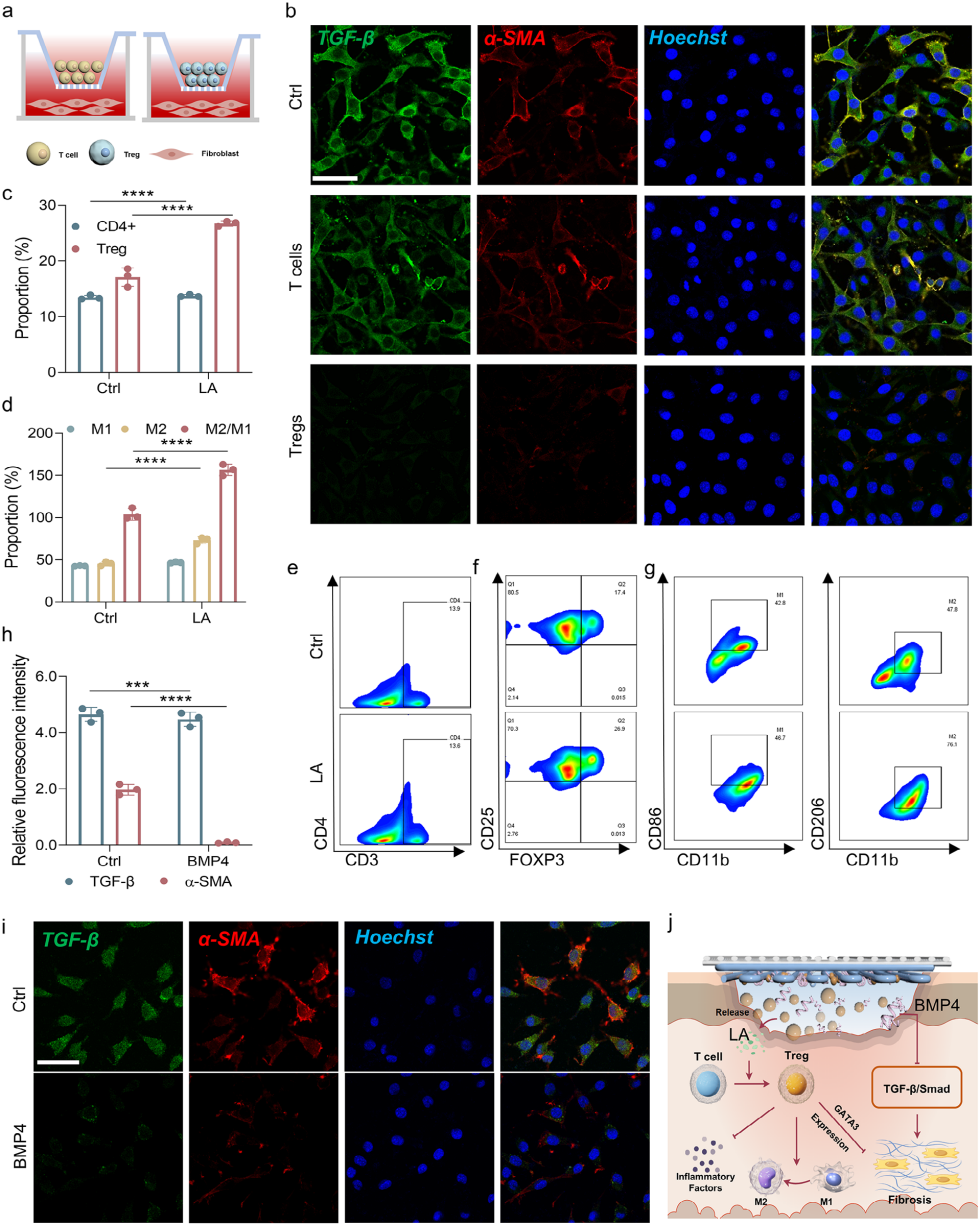
Validation of the LA‐mediated regulation of the immune environment and inhibition of fibrosis by Tregs and BMP4 proteins. (a) Schematic diagram of the cell culture process. (b) Representative fluorescence images of MFBs and fibrillation marker expression. Scale bars: 50 µm. (c) Quantification of the T‐cell flow assay results. (d) Quantification of the results of the macrophage flow analysis. (e) Representative flow cytometry results of LA‐regulated CD4^+^ T‐cell polarization. (f) Representative flow cytometry results of LA‐regulated Treg cell polarization. (g) Representative flow plots of LA‐regulated macrophage polarization. (h) Quantitative analysis of the TGF‐β/a‐SMA expression levels. (i) Representative fluorescence images of MFBs and fibrillation marker expression. Scale bars: 50 µm. (j) Schematic diagram of the process by which LA and BMP4 inhibit fibrosis. All the data were generated from at least three independent experiments and are presented as the means ± SDs. The P values were calculated via one‐way analysis of variance (ANOVA). *P < 0.05, **P < 0.01, ***P < 0.001, and ****P < 0.0001.

In a range of normal and pathological immune environments, Tregs play pivotal roles in modulating immune responses, maintaining immune tolerance, and preventing excessive inflammation. Tregs in the skin serve as crucial regulators of FB activation and play a vital role in controlling dermal fibrosis. Specifically, the transcription factor GATA3, which is expressed in Tregs, has been shown to inhibit FB activation and the progression of dermal fibrosis. During the inflammatory phase of wound healing, Tregs mitigate the exacerbation of fibrosis induced by the excessive production of inflammatory cytokines. This is achieved through the secretion of anti‐inflammatory cytokines such as IL‐4 and IL‐10, which help to modulate the inflammatory response [13]. To further investigate the potential of Tregs in alleviating fibrosis, we simulated the inflammatory wound microenvironment by stimulating FBs with lipopolysaccharide (LPS) and coculturing them with Tregs and T cells in a Transwell system (Figure 2a). The coculture setup allowed us to examine the interactions between immune cells and FBs under conditions that mimic the inflammatory phase of wound healing. Compared with those in the Ctrl group, the expression of key markers associated with fibrosis, including TGF‐β and alpha‐smooth muscle actin (α‐SMA), was significantly lower in FBs cocultured with Tregs than in those cocultured with T cells (*P* < 0.0001) (Figure 2b; Figure S1). We measured the concentrations of the anti‐inflammatory cytokines IL‐4 and IL‐10 in the lower chamber of the coculture system. Compared with those in the Ctrl group, the levels of these cytokines were significantly greater in both the T‐cell and Treg groups, indicating that the simulated inflammatory environment activated the immune response of T cells. Notably, the concentrations of IL‐4 and IL‐10 secreted by Tregs were significantly greater than those secreted by T cells (Figures S2 and S3), suggesting that Tregs can produce high levels of anti‐inflammatory cytokines during inflammation to suppress inflammatory responses. These findings demonstrate that Tregs can effectively suppress the differentiation of FBs into MFBs in an inflammatory environment, thereby inhibiting the fibrotic process. This study provides compelling evidence that Tregs play a protective role in regulating FB function and limiting the pathological progression of fibrosis in the context of wound healing. The ability of Tregs to modulate FB activation and prevent excessive fibrosis underscores their therapeutic potential in conditions characterized by chronic inflammation and fibrosis, suggesting a promising avenue for future interventions in tissue repair and regenerative medicine.

However, efficiently and controllably modulating the polarization of Tregs at the wound site presents a significant challenge. Previous studies have shown that Tregs are excessively expressed and accumulate in tumor tissues, severely limiting the efficacy of tumor immunotherapy. The primary reason for this is the excessive LA produced by tumor glycolysis, which efficiently polarizes Tregs [14]. Therefore, in this study, we first confirmed that LA can induce the polarization of T cells into Tregs in vitro. Mouse spleen‐derived T cells were used to assess the effects of LA on Treg polarization. T cells were isolated from 4‐week‐old mice and subsequently stimulated with 10 mm LA for a period of three days. Flow cytometric analysis revealed an increase in the proportion of CD4^+^ T cells upon LA stimulation (*P* < 0.0001) (Figure 2e). Notably, within the CD4^+^ T‐cell population, the percentage of Tregs (characterized as CD25^+^ and FOXP3^+^) was markedly elevated (*P* < 0.0001) (Figure 2c,f; Figure S4). These findings suggest that LA not only enhances the overall polarization of CD4^+^ T cells but also specifically favors the differentiation of CD4^+^ T cells into Tregs. To further investigate the impact of the aforementioned metabolic preference on cellular metabolic characteristics, we compared the glucose and LA uptake capacities between Tregs and conventional T cells. Consistent with their known metabolic properties [15], Tregs presented a significantly lower glucose uptake capacity than conventional T cells did at all time points (Figure S5), whereas their LA uptake was markedly greater (Figure S6). Additionally, we examined the proliferative response of Tregs under LA stimulation and found that, compared with glucose‐stimulated Tregs, Tregs preferentially rely on LA as a key metabolic substrate to support their proliferation (Figure S7). These findings indicate that LA promotes Treg polarization through mechanisms involving metabolic reprogramming. By serving as an alternative energy source that Tregs utilize more efficiently than conventional T cells do, LA enhances their survival and proliferative capacity. Furthermore, LA supports the functional stability of Tregs and facilitates an immunosuppressive microenvironment, thereby promoting their differentiation and maintenance. As an endogenous ligand of the GPR81 receptor, LA binds to GPR81 expressed on the T‐cell surface, reducing intracellular cAMP levels and thereby modulating T‐cell activation thresholds and functional responses [16]. Activation of GPR81 signaling not only helps maintain an immunosuppressive microenvironment but also enhances the production of anti‐inflammatory factors such as IL‐10, further reinforcing Treg functionality. In summary, LA‐induced Treg polarization is a synergistic multimechanism process: LA acts directly as both a metabolic substrate and a signaling molecule to promote Treg proliferation, function, and stability while also indirectly facilitating Treg polarization by fostering an immunosuppressive microenvironment conducive to Treg differentiation [17, 18].

In addition to Tregs, macrophages, which are abundant in the wound microenvironment, are crucial for regulating immune responses and tissue repair [19, 20, 21]. To evaluate the impact of LA on macrophage polarization, we utilized bone marrow‐derived macrophages (BMDMs) in this study. Prior to LA stimulation, macrophages are activated to the M1 phenotype via LPS, mimicking the inflammatory phase of wound healing. Following LA treatment, flow cytometric analysis revealed a significant reduction in the CD86^+^ population, indicative of M1 macrophages, while the CD206^+^ population, representing M2 macrophages, increased significantly. Furthermore, the M2/M1 macrophage ratio substantially increased (*P* < 0.0001) (Figure 2d,g; Figure S8). These results provide strong evidence that LA promotes the polarization of proinflammatory M1 macrophages toward the anti‐inflammatory M2 phenotype, suggesting its potential to favor the resolution of inflammation during wound healing. Together, these findings highlight the dual effects of LA in the wound immune microenvironment: enhancing the polarization of Tregs and promoting the M2 macrophage phenotype. These findings suggest that LA could play a significant role in modulating the immune response during tissue repair, potentially offering therapeutic avenues for controlling inflammation and promoting tissue regeneration in chronic wounds.

Furthermore, BMP4 has been shown to suppress the production of ECM proteins induced by TGF‐β1 in pulmonary FBs. In the process of fibrosis, TGF‐β serves as the central profibrotic driver, promoting the differentiation of FBs into MFBs by activating the canonical Smad signaling pathway, which directly initiates the expression of genes such as α‐SMA and collagen. In contrast, BMP4 acts as a pivotal antagonist that counteracts this process by activating its own Smad1/5/8 pathway, competing for the common Smad4 cofactor, and upregulating the expression of inhibitory Smad7. Smad7 directly abrogates TGF‐β receptor signaling, thereby suppressing its profibrotic activity at the source and effectively inhibiting MFB differentiation and tissue fibrosis [22]. Building upon these findings, the present study aims to explore the potential of BMP4 in inhibiting the FBs‐to‐MFBs transition during wound healing, thereby reducing fibrosis and minimizing scar formation. In this study, FBs were cultured in vitro and stimulated with TGF‐β1 to induce MFB differentiation. The BMP4 protein was then cocultured in medium to assess its impact on FB transformation. Immunofluorescence analysis revealed a significant reduction in the expression of α‐smooth muscle actin (α‐SMA), a hallmark marker of MFBs, in the BMP4‐treated group (*P* < 0.0001) (Figure 2h,i). This reduction suggests that BMP4 effectively inhibits the differentiation of FBs into MFBs, a process that is critical in the pathogenesis of fibrosis and scar formation. MFBs are a central feature of scar tissue, where they secrete large quantities of ECM proteins, contributing to the development of fibrotic tissue [23]. By preventing the differentiation of FBs into MFBs, BMP4 can substantially reduce the accumulation of ECM and mitigate scar formation. These findings highlight the potential of BMP4 as a therapeutic agent for controlling fibrosis during wound healing, offering innovative avenues for improving tissue repair and minimizing scarring (Figure 2j).

### 2.2 Fabrication and Characterization of the PLGA@LA‐BMP4‐PG Scaffold

**FIGURE 3:**
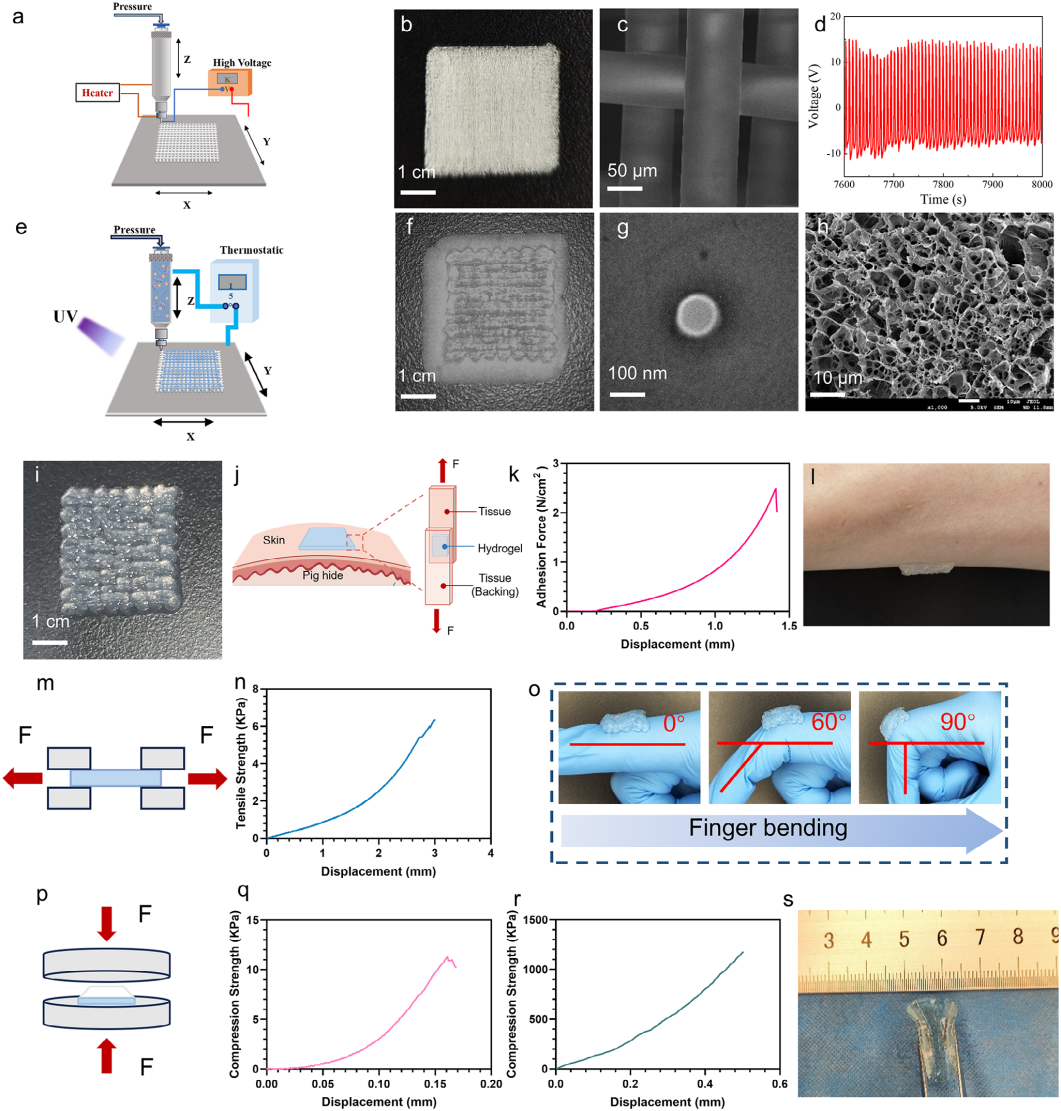
Preparation and characterization of PLGA@LA‐BMP4‐PG. (a) Schematic diagram of PLA scaffold preparation. (b) Images of PLA scaffolds. Scale bar: 1 cm. (c) SEM image of PLA. Scale bar: 50 µm. (d) The piezoelectric properties of the PLA scaffolds were analyzed. (e) Schematic diagram of the PLA‐GelMA scaffold preparation. (f) Images of the PLA‐GelMA hydrogels. Scale bar: 1 cm. (g) TEM images of PLGA nanoparticles. Scale bar: 100 nm. (h) SEM image of the GelMA hydrogel. Scale bar: 10 µm. (i) Images of GelMA hydrogels. Scale bars = 1 cm. (j) Schematic of the GelMA adhesion test. (k) Adhesion properties of GelMA. (l) Demonstration of the adhesion of GelMA to human skin tissue. (m) Schematic diagram of the GelMA tensile strength test. (n) Tensile strength of GelMA. (o) Adaptive stretching of GelMA at the joints. (p) Schematic diagram of the GelMA and PLA compression strength tests. (q) Compression strength of GelMA. (r) Compression strength of PLA. (s) Adaptive compressive strength profile of GelMA. The data are presented as the means ± SDs. The P values were calculated via one‐way analysis of variance (ANOVA). *P < 0.05, **P < 0.01, ***P < 0.001, and ****P < 0.0001.

Following the identification of two key functional substances, we proceeded with the design of a smart wound scaffold patch. PLA was selected because of its excellent piezoelectric properties, which enable the generation of an electric field in response to mechanical stress exerted by the surrounding tissues during wound healing [24]. In this study, PLA was fabricated via a 3D printer under electrostatic printing conditions (Figure 3a), resulting in a dense grid scaffold that provides a 3D structure to facilitate cell attachment and migration. This configuration not only promotes oxygen diffusion to the wound site but also mitigates the risk of hypoxia‐induced tissue deterioration. The printed PLA scaffold exhibited a filament diameter of approximately 50 µm (Figure 3b,c) and generated an electrical voltage of approximately 20 V (Figure 3d), underscoring its potential for dynamic interaction with the wound microenvironment. Additionally, to determine whether the PLA scaffold could effectively generate an electric field in a dynamic environment, we measured its output voltage under various external forces. As shown in Figure S9, the PLA scaffold effectively generated an electric field within the applied force range of 0.01 to 0.10 N. Furthermore, the output voltage increased proportionally with increasing applied force. GelMA, a biocompatible photocross‐linkable hydrogel, was chosen for the sublayer of the scaffold because of its versatility as a drug delivery system. In this study, GelMA was printed in situ onto the PLA scaffold via a 3D extrusion printer (Figure 3e), followed by ultraviolet (UV) photocross‐linking to create a bilayer grid scaffold (Figure 3f). The resulting porous GelMA structure (Figure 3h,i) facilitates cell migration and proliferation, enhancing the regenerative capacity of the scaffold. The bilayer scaffold, with GelMA forming the bottom layer and PLA positioned at the top, allows for the controlled and sustained release of therapeutic agents, which helps alleviate inflammation and fibrosis during wound healing.

To enable the effective loading and controlled release of LA, PLGA nanoparticles (PLGA@LA NPs) with a diameter of approximately 100 nm were fabricated as carriers for LA (Figure 3g; Figure S10). PLGA is an FDA‐approved biomaterial known for its favorable degradation profile and excellent biosafety, as its degradation products—LA and glycolic acid (GA)—are naturally occurring metabolic byproducts. PLGA degradation is accelerated in acidic environments, which triggers the release of LA and reduces the tissue pH, thereby promoting PLGA degradation via a positive feedback loop. To further enhance the therapeutic potential of this scaffold, GelMA scaffolds loaded with PLGA and BMP4 were printed in situ on PLA scaffolds. This multifunctional approach integrates both anti‐inflammatory and antifibrotic therapies into the wound healing microenvironment. To evaluate the drug release and degradation behavior of the bilayer scaffold, the concentration of LA released at different time points was continuously monitored in an in vitro environment. The results demonstrated that during the initial degradation phase (0–3 days), the LA release rate was relatively slow, which was attributed primarily to the preliminary hydrolysis of the superficial PLGA layer, resulting in the release of only a small amount of LA (Figure S11). Upon entering the intermediate degradation stage (3–7 days), the LA release rate increased markedly, indicating a burst release of LA from the PLGA@LA complex around day 3. The released LA further accelerated the hydrolysis of the ester bonds in PLGA, leading to an enhanced degradation effect. In the later degradation stage (7–10 days), as the remaining degradable material within the scaffold was gradually depleted, the LA release rate slowed significantly and eventually stabilized. Furthermore, the LA concentration in the soaking solution was approximately 10 mM on day 10, confirming that the scaffold maintained its structural integrity throughout the release period and avoided uncontrolled degradation caused by the accumulation of acidic products. To more intuitively observe the degradation rate of the PLA scaffold under such acidic conditions, we measured its weight loss at selected time points. Owing to its strong hydrophobicity, the PLA scaffold degraded slowly even in the low‐pH environment during the middle‐to‐late stages, with a weight loss of approximately 29% by day 10 (Figure S12). These findings collectively demonstrated that the degradation process of the PLGA@LA‐BMP4‐PG scaffold exhibited a controlled degradation pattern, with its positive feedback effect clearly confined within the anticipated range.

To further assess the biosafety of the degraded products, we collected soaking solutions from the scaffold at representative time points (days 3, 7, and 10) and used them to culture FBs. CCK‐8 assay analysis revealed no reduction in cell viability compared with that of the Ctrl group, confirming that the acidic microenvironment and degradation products generated by the PLGA@LA‐BMP4‐PG scaffold possess favorable biosafety (Figure S13). To evaluate the potential effects of scaffold degradation products on cell proliferation at different time points, we examined the proliferation of FBs via an EdU staining assay. The results revealed that (Figure S14) compared with those in the Ctrl group, FBs treated with degradation products collected on day 3 exhibited significant promotion of proliferation. This pro‐proliferative effect was also observed in the groups treated with the scaffold immersion solutions obtained on days 7 and 10, indicating that the compounds released during the early to middle stages of scaffold degradation can effectively increase the cell proliferation capacity. LA can serve as an energy substrate to partially support cell growth, which is beneficial for wound healing. To further investigate the immunomodulatory effects of the scaffold degradation products, we analyzed their impact on Treg polarization via flow cytometry. Compared with those collected from the Ctrl group, the extracts collected at all three time points demonstrated an increased capacity to promote Treg polarization (Figures S15 and S16). Notably, extracts from the intermediate and late stages exhibited a significantly greater ability to induce Treg polarization than did those from the early stage. This observation is consistent with the design concept of the scaffold, which aims to accelerate LA release during the inflammatory phase, suggesting its potential to effectively induce an anti‐inflammatory microenvironment conducive to the resolution of inflammation and the promotion of tissue regeneration. This process allows the release of LA to align with the varying needs of the wound healing stages: a lower release rate during the early healing phase to avoid excessive acidity that could inhibit cell proliferation and a higher release rate in the later inflammatory phase to reduce inflammation through Treg polarization and inhibit fibrosis.

The LA concentration and GelMA mass loss rate results indicate that the electric field generated by the PLA scaffold promotes drug release from GelMA (Figures S17 and S18). On Day 10, the LA release concentration in the PLGA@LA‐BMP4‐G group (without the upper PLA scaffold) was approximately 7.2 mm. Under PLA electric field stimulation, the LA release concentration increased to 9.3 mm. Concurrently, the mass loss rate of GelMA also increased from approximately 72% to 83%. The applied electric field generated by the piezoelectric PLA layer enhances drug release from the GelMA hydrogel, primarily through the combined action of electroosmotic flow and induced changes in the hydrogel nanostructure. The GelMA network, which is negatively charged, establishes a fixed charge density within its pores. Upon the application of an electric field, mobile cations in the hydration layer are driven toward the cathode, creating a bulk fluid movement known as electroosmotic flow that actively convects dissolved drug molecules through the hydrogel's aqueous channels, significantly augmenting their passive diffusion. Concurrently, the electric field exerts an electrostatic repulsive force on the anionic polymer chains of the GelMA matrix. This force transiently swells the hydrogel network, increasing its mesh size and porosity, which in turn reduces the diffusion barrier for the drug molecules and facilitates their more rapid egress from the polymer matrix. These two mechanisms operate synergistically, with electroosmosis providing a convective pumping force and an enlarged pore structure lowering the intrinsic resistance to drug transport, thereby collectively accounting for the observed acceleration in drug release.

In addition to the biological functions of the scaffold, we evaluated the mechanical properties of both the PLA and GelMA scaffolds. The adhesive strength of the GelMA grid scaffold was tested using porcine skin tissue, which serves as a model for human skin. The hydrogel adhered to two pieces of skin, which were subjected to opposite directional forces until detachment occurred (Figure 3j). The results demonstrated that the adhesive strength of GelMA was 2.5 N/cm^2^ (Figure 3k), indicating its strong adhesion to skin tissues. This adhesion was further confirmed by inverting the scaffold on human skin tissue, which showed excellent adhesion (Figure 3l). The tensile properties of the GelMA scaffold were also assessed by stretching the material at a uniform rate until rupture (Figure 3m). The tensile strength of GelMA was approximately 5 kPa (Figure 3n), demonstrating its ability to withstand mechanical stress during daily activities. Additionally, when the GelMA scaffold was applied to a knuckle joint and subjected to movement, it exhibited remarkable tensile flexibility, adapting to the dynamic movements of the joint without compromising structural integrity (Figure 3o). Furthermore, we tested the compressive properties of both the PLA and GelMA scaffolds (Figure 3p). The GelMA scaffold exhibited a compressive strength of approximately 11 kPa, whereas the PLA scaffold demonstrated a compressive strength of approximately 1200 kPa (Figure 3q,r). Importantly, the hydrogel layer withstood compression and bending forces without fracturing (Figure 3s), confirming the scaffold's ability to maintain its structural integrity under everyday mechanical forces during the treatment process.

### 2.3 Analysis of Biocompatibility, Cell Migration, and Angiogenesis In Vitro

**FIGURE 4:**
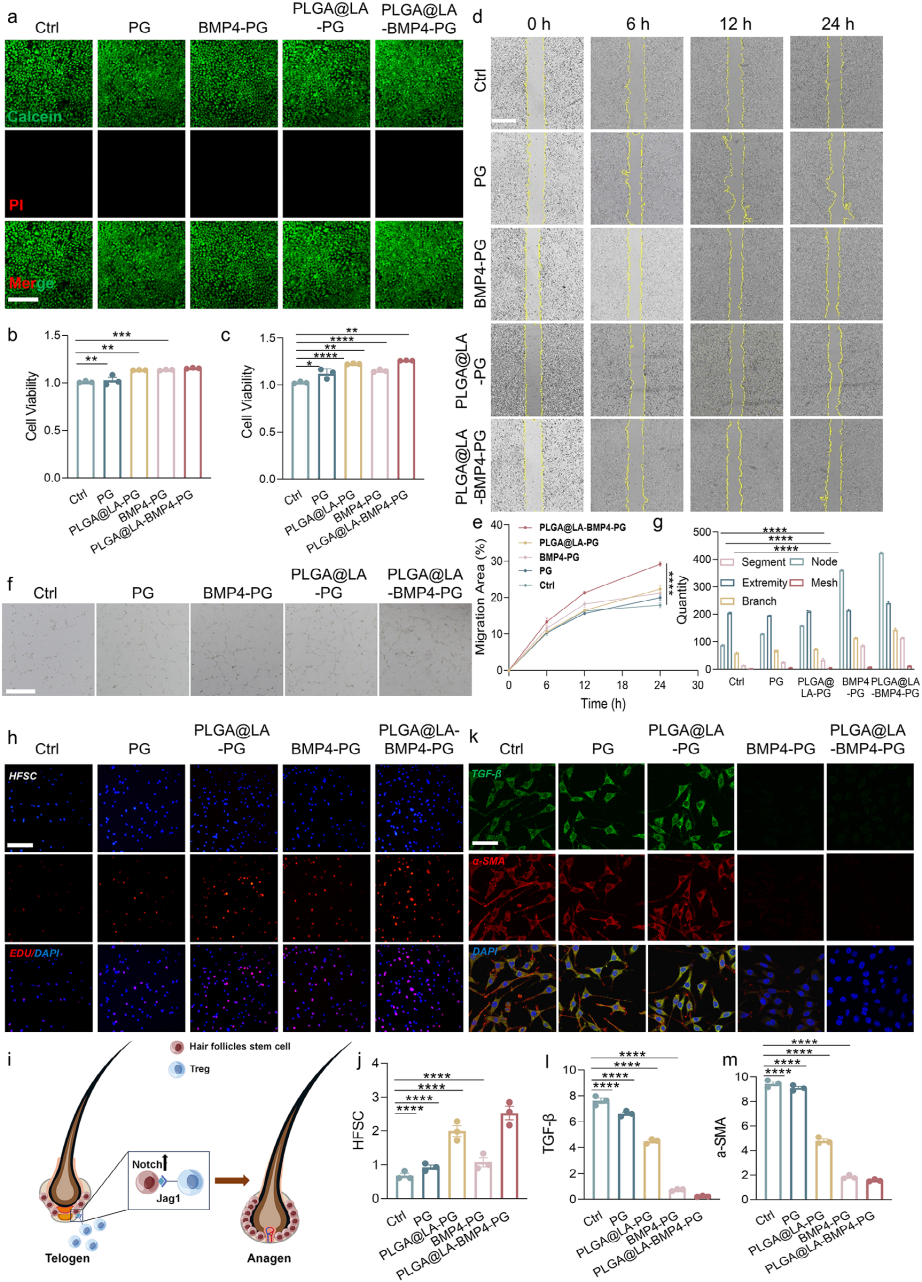
Evaluation of wound healing and antiscar processes in vitro. (a) Calcein AM/PI fluorescence staining was used to examine live/dead cells. Scale bar: 50 µm. (b) CCK8 results of cell proliferation on day 1. (c) CCK8 results of cell proliferation on day 3. (d) Scratch healing experiments on keratinocytes subjected to different pretreatment conditions at 0, 6, 12, and 24 h. Scale bar: 200 µm. (e) Quantitative analysis of the results of the scratch healing experiments. (f) Tube formation ability of HUVECs subjected to different treatments. Scale bar: 200 µm. (g) Quantitative results of tube formation experiments. (h) Fluorescence staining plots of EdU‐labeled proliferating HFSCs. Scale bar: 100 µm. (i) Diagram of the mechanism by which Tregs promote HF regeneration. (j) Quantitative results of EdU‐labeled proliferating HFSCs. (k) Representative fluorescence images of MFBs and fibrillation marker expression. Scale bars: 50 µm. (l,m) Quantitative analysis of the fluorescence intensity of TGF‐β and α‐SMA. All the data were generated from at least three independent experiments and are presented as the means ± SDs. The P values were calculated via one‐way analysis of variance (ANOVA). *P < 0.05, **P < 0.01, ***P < 0.001,s and ****P < 0.0001.

We evaluated the biocompatibility of the bilayer scaffold by analyzing its effects on keratinocyte survival via live/dead cell staining and CCK‐8 assays. The live/dead staining results (Figure 4a) revealed high cell viability in all the groups, including the blank PG (PLA‐GelMA) scaffold group, confirming the excellent biosafety of the materials and delivery carriers used in this study. This observation suggests that both PLA and GelMA, as well as the fabrication process, are nontoxic and conducive to cell survival. Interestingly, the PG scaffold group presented greater cell viability than the Ctrl group did, indicating that the electric field generated by the piezoelectric properties of PLA may stimulate keratinocyte proliferation. The electric field enhances cellular behaviors such as migration, proliferation, and alignment, which are crucial for efficient wound healing [25]. These findings underscore the potential of the piezoelectric PLA scaffold to actively contribute to tissue regeneration beyond serving as a structural support. Moreover, cell viability in the BMP4‐PG and PLGA@LA‐PG groups was significantly greater than that in both the Ctrl and PG groups, suggesting that the addition of BMP4 and LA further enhanced cell survival and proliferation. BMP4, a well‐established growth factor, likely promotes cell proliferation and survival through its role in activating Smad‐dependent pathways that regulate keratinocyte growth and differentiation [26]. Moreover, LA, which is delivered via PLGA nanoparticles, may provide additional benefits via lactate dehydrogenase (LDH), in which LA is converted to pyruvate throughLDH and then enters the mitochondria, where it participates in the tricarboxylic acid (TCA) cycle. This metabolic pathway enhances the production of energy by facilitating the metabolism of nutrients, thereby promoting cell proliferation [27]. The results of the CCK‐8 assay corroborated the live/dead staining findings, which revealed a similar trend. On days 1 and 3 posttreatment, the PG scaffold group maintained high cell viability, whereas the groups containing BMP4 and PLGA@LA (BMP4‐PG and PLGA@LA‐PG) presented significantly greater cell survival rates than did the blank PG scaffold and Ctrl groups (Figure 4b,c). These findings further validated the synergistic effects of the piezoelectric properties of PLA and the bioactive molecules BMP4 and LA on promoting keratinocyte survival and proliferation. These results highlight the superior biocompatibility and multifunctionality of the bilayer scaffold. The combination of a piezoelectric PLA scaffold and a bioactive GelMA hydrogel not only supports keratinocyte survival but also actively promotes wound healing by leveraging the electric field effects of PLA, the growth‐promoting properties of BMP4, and the inflammation‐modulating role of LA. Together, these mechanisms offer a promising strategy for developing next‐generation wound healing scaffolds capable of addressing the multifaceted challenges of tissue repair.

The migration speed of skin cells directly influences the wound healing rate. To further evaluate the ability of the bilayer scaffold to promote cell migration, we conducted scratch assays and assessed the migratory capacity of the cells under different experimental conditions. The results revealed that after 24 h, the scratch closure rates increased across all groups, but significant differences were observed between the groups (Figure 4d,e). In the Ctrl group, the scratch closure rate was relatively low, indicating limited cell migration in the absence of scaffold support or bioactive factors. In contrast, the PG group presented a significantly greater scratch closure rate than the blank Ctrl group did, suggesting that the PG scaffold could effectively enhance cell migration. This improvement is attributed to the piezoelectric properties of PLA, where the generated microelectric field activates signaling pathways that promote cell migration and proliferation, thereby accelerating scratch closure. The primary mechanism by which an electric field promotes cell migration is “electrotaxis”; the fundamental principle is that cells can sense an external electric field and undergo directed migration [28]. Specifically, the weak electric field generated by the PLA scaffold is anticipated to induce an asymmetric redistribution of charged receptors (such as growth factor receptors and integrins) on the cell membrane, thereby establishing front‐rear polarity within the cell. This polarity subsequently activates key signaling pathways, including the PI3K/Akt pathway, and leads to the spatially polarized activation of proteins that orchestrate cell movement (e.g., Rac and Cdc42, which promote protrusion formation at the front, and RhoA, which mediates contraction at the rear). Ultimately, this cascade of intracellular events drives the directional reorganization of the cytoskeleton: actin polymerization and lamellipodia formation at the cell front, coupled with contraction at the rear, resulting in directed migration along the field. Moreover, the scratch closure rates were significantly greater in the BMP4‐PG and PLGA@LA‐PG groups than in both the Ctrl and PG groups (*P* < 0.01). Over time, the BMP4‐PG and PLGA@LA‐PG groups demonstrated a synergistic effect on scratch closure, achieving significantly higher rates of closure than the PG group alone at 24 h. These findings underscore the importance of incorporating functional factors such as BMP4 and LA to increase the ability of the scaffold to promote cell migration.

Angiogenesis plays a pivotal role in tissue regeneration, particularly in wound healing, as it ensures the delivery of the oxygen and nutrients necessary for the functional restoration of skin tissue. To evaluate the angiogenic potential of the bilayer scaffold, an in vitro tube formation assay was conducted to assess the ability of vascular endothelial cells to form capillary‐like structures. Compared with that in the Ctrl group, the tube‐forming ability of endothelial cells in the BMP4‐treated group was significantly greater (Figure 4f,g). This finding aligns with previous research showing that BMP4 promotes endothelial cell proliferation and migration by activating Smad‐dependent and other angiogenesis‐related pathways, thereby facilitating vascular regeneration [29]. The improved tube formation observed in the presence of BMP4 suggests that its incorporation into the bilayer scaffold could actively contribute to the revascularization of the wound site. Enhanced angiogenesis not only accelerates tissue regeneration by supplying essential nutrients and oxygen but also supports the recruitment of other cell types involved in the repair process, such as FBs and keratinocytes. Moreover, the piezoelectric properties of the scaffold may provide an additional stimulatory effect by promoting cellular alignment and enhancing cell‐to‐cell communication, further supporting angiogenesis and wound healing. In conjunction with the previously discussed cell proliferation and migration results, the angiogenic effects of BMP4 within the bilayer scaffold reinforce its multifunctionality. The combined effects of enhanced endothelial cell activity, keratinocyte proliferation, and FB modulation contribute to a holistic approach to tissue regeneration. Additionally, the controlled release of LA from PLGA nanoparticles likely creates a favorable microenvironment for angiogenesis by modulating local pH levels and reducing inflammation, which is essential for efficient capillary network formation.

### 2.4 Promotion of the Proliferation of HFSCs and L929 Cells and Inhibition of Fibrosis by PLGA@LA‐BMP4‐PG In Vitro

Hair follicle stem cells (HFSCs) are integral to the wound healing process because of their ability to migrate to the site of injury and differentiate into keratinocytes, which form a new epidermis, and into FBs or endothelial cells, which contribute to dermal regeneration [30]. The regeneration of hair follicles is particularly critical for the functional restoration of skin tissue, as it is closely associated with the regeneration of sebaceous glands. In this study, we investigated the effects of Treg‐conditioned media on HFSCs in vitro for 1 and 3 days. The results revealed that Tregs significantly promoted the proliferation of HFSCs (Figure S19), which aligns with the essential role of Tregs in supporting tissue regeneration [7]. FBs are the primary proliferating cells during wound healing, actively participating in tissue repair by filling the defect and contributing to ECM formation. The proliferation rate is directly correlated with the speed of wound healing. HFSCs, as resident stem cells of the skin, not only contribute to the regeneration of epidermal layers but also aid in dermal repair through their differentiation into FBs and other skin cells. In this context, we evaluated the ability of the bilayer scaffold to promote the proliferation of both HFSCs and FBs via the EdU labeling method. When HFSCs and T cells were cocultured and pharmacologically stimulated, EdU staining (red) revealed that the bilayer scaffold facilitated HFSC proliferation by releasing LA‐polarized T cells, which in turn promoted Treg polarization (Figure 4h,j) [31]. In parallel, the results of the FB proliferation assay were similar, confirming that the bilayer scaffold not only enhanced HFSC proliferation but also promoted FB proliferation (Figures S20 and S21). The mechanism may involve the proliferation and differentiation of hair follicle stem cells through the Notch signaling pathway via high Treg expression of Jag1 (Figure 4i).

Scar formation is driven predominantly by the differentiation of FBs into MFBs, a process that leads to the excessive synthesis and secretion of ECM proteins. The accumulation of ECM results in tissue fibrosis, making the prevention of FBS overactivation a critical strategy for mitigating scar formation. TGF‐β is a key regulatory factor in this process, as it drives the differentiation of FBs into MFBs. MFBs, characterized by the expression of α‐smooth muscle actin (α‐SMA), play a central role in scar formation. In this study, we analyzed the expression of TGF‐β and α‐SMA as markers to evaluate the antifibrotic potential of the bilayer scaffold at the cellular level.

To model the proliferative phase of wound healing, FBs were induced to differentiate into MFBs via TGF‐β stimulation, simulating the profibrotic environment of a healing wound. Simultaneously, FBs were treated with different drug‐loaded scaffolds to assess their antifibrotic effects. Immunofluorescence staining was used to measure the expression of TGF‐β and α‐SMA, providing insights into the ability of the scaffold to modulate fibrosis. Compared with those in the Ctrl group, the FBs in the bilayer scaffold group presented significantly lower expression of TGF‐β and α‐SMA (Figure 4k–m), indicating a marked reduction in MFB differentiation. These findings suggest that LA, which is delivered through PLGA nanoparticles, indirectly mitigates fibrosis by polarizing Tregs, which in turn regulate the inflammatory environment and suppress profibrotic signaling pathways. This mechanism highlights the scaffold's ability to modulate immune responses and prevent FB overactivation. Among the treatment groups, the PLGA@LA‐BMP4‐PG scaffold displayed the most pronounced antifibrotic effects, resulting in a synergistic reduction in both TGF‐β and α‐SMA expression. Notably, the BMP4‐PG group presented stronger antifibrotic activity than the PLGA@LA‐PG group did, suggesting that BMP4 directly inhibits fibrosis by suppressing critical profibrotic signaling pathways, likely through its modulation of Smad‐dependent transcriptional activity. The combination of BMP4 and LA in the bilayer scaffold appears to leverage distinct yet complementary mechanisms: BMP4 directly targets fibrosis‐related pathways, whereas LA modulates the immune microenvironment to further inhibit fibrosis. These results underscore the potential of the bilayer scaffold to serve as a multifunctional therapeutic platform for wound healing. By integrating immune modulation and direct pathway inhibition, the scaffold offers a promising strategy for controlling fibrosis, reducing scar formation, and promoting functional tissue regeneration. The combined delivery of BMP4 and LA represents a highly innovative approach for addressing the complex challenges of wound healing and fibrosis management.

### 2.5 Analysis of Tregs and Macrophage Polarization by PLGA@LA‐BMP4‐PG In Vitro

Our cellular‐level investigations simulating the inflammatory and proliferative phases of wound healing revealed that Tregs exhibit significant antifibrotic effects. During the inflammatory phase, Tregs mitigate fibrosis by releasing anti‐inflammatory cytokines and suppressing proinflammatory cytokine production. In the proliferative phase, they directly inhibit FB hyperactivation and ECM deposition through specific functional transcription factors. Although fibrosis predominantly develops during the proliferation and remodeling phases, the inflammatory phase serves as the critical initiation point for fibrotic processes. The inflammatory response in wound healing acts as a key regulatory node driving tissue repair and fibrosis through complex cellular and molecular networks [32].

The fibrotic initiation mechanism during inflammation involves sequential events: inflammatory cells release signaling molecules that activate FBs, triggering their transformation into MFBs, which subsequently secrete ECM components to form scar tissue. The cellular basis of inflammation‐initiated fibrosis primarily involves neutrophil infiltration and macrophage polarization dynamics. Neutrophils rapidly infiltrate injury sites during inflammation, releasing ROS and matrix metalloproteinases (MMP‐8/9) to clear necrotic tissue and pathogens while simultaneously inducing FB activation. M1 macrophages (proinflammatory phenotype) initiate inflammatory cascades through the secretion of IL‐6, IL‐1β, and TNF‐α, which promote the differentiation of local FBs and circulating fibrocytes into contractile MFBs via RhoA/ROCK pathway activation, accompanied by collagen deposition to form a provisional matrix [2].

**FIGURE 5:**
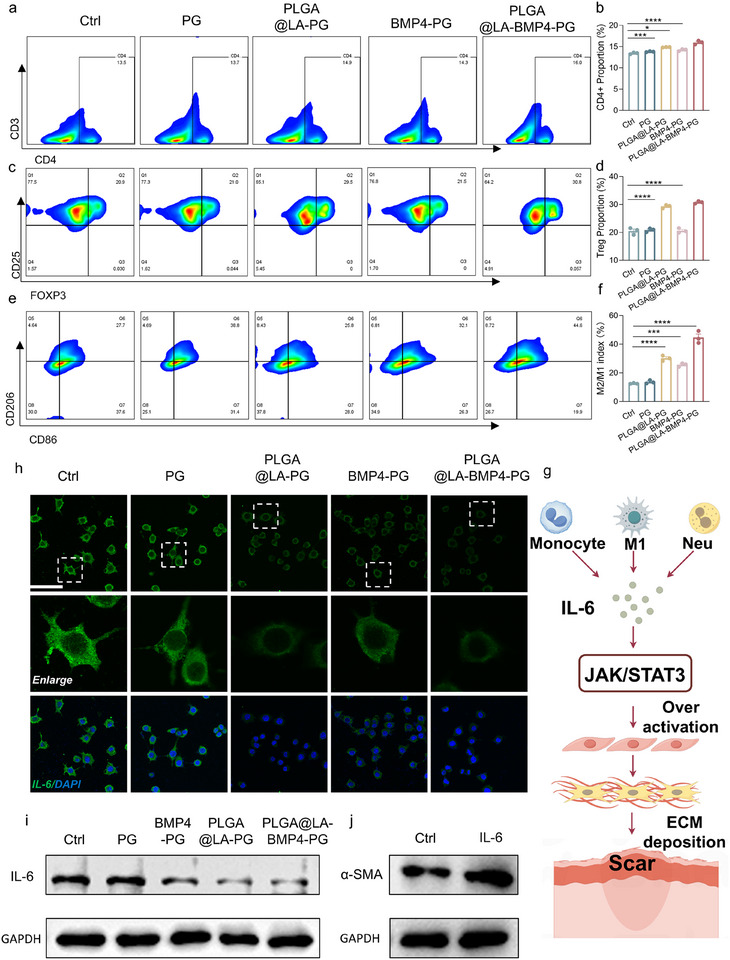
Tregs inhibit fibrosis by reducing the level of IL‐6. (a) Representative plots of the flow analysis results of CD4^+^ T‐cell polarization. (b) Quantification of the CD4^+^ T‐cell flow cytometry results. (c) Representative plots of the flow analysis results of Treg polarization. (d) Quantification of the Treg flow cytometry results. (e) Representative plots of the results of flow cytometry analysis of macrophage polarization. (f) Quantification of the results of the macrophage flow assay. (g) Schematic representation of the mechanism by which IL‐6 exacerbates fibrosis. (h) Representative fluorescence images of IL‐6 expression in macrophages. Scale bars: 50 µm. (i) Western blot analysis of IL‐6 in macrophages. (j) Western blot of α‐SMA in FBs. All the data were generated from at least three independent experiments and are presented as the means ± SDs. The P values were calculated via one‐way analysis of variance (ANOVA). *P < 0.05, **P < 0.01, ***P < 0.001, and ****P < 0.0001.

In our experimental system, LA‐induced Treg polarization was identified as the principal mechanism underlying antiscarring and pro‐healing effects. Using murine splenic T cells as targets subjected to various treatments, flow cytometry analysis demonstrated that both the PLGA@LA‐PG and PLGA@LA‐BMP4‐PG groups presented significantly greater CD4^+^ T‐cell polarization than the control group did (Figure 5a,b; Figure S22). CD4^+^ T lymphocytes, crucial immune regulators, orchestrate repair initiation by facilitating pathogen clearance while preventing excessive inflammation and wound deterioration. Notably, both treatment groups effectively polarized Treg populations (Figure 5c,d), confirming that our core‐shell‐structured PLGA@LA nanoparticles successfully achieved controlled LA release, confirming the feasibility of PLGA encapsulation for sustained release‐mediated Treg polarization enhancement.

To elucidate Treg‐mediated macrophage regulation during inflammation, we employed a Transwell coculture system containing LPS‐stimulated M0 macrophages and scaffold‐treated T cells. Flow cytometric analysis revealed that the Treg‐polarizing PLGA@LA‐PG and PLGA@LA‐BMP4‐PG groups significantly promoted M2 macrophage polarization (Figure 5e,f; Figure S23), with M2/M1 ratios substantially exceeding the control levels. This finding demonstrates the capacity of Tregs to suppress proinflammatory M1 polarization while enhancing anti‐inflammatory M2 differentiation [33]. Intriguingly, the BMP4‐PG group also presented elevated M2/M1 ratios, which was attributable to the dual antifibrotic and anti‐inflammatory properties of BMP4, which facilitate M2 polarization [34]. This macrophage polarization modulation not only directly reduces proinflammatory cell populations at the cellular level but also establishes a foundation for the subsequent suppression of proinflammatory cytokine production and the mitigation of fibrotic progression.

### 2.6 Analysis of Fibrosis Suppression by PLGA@LA‐BMP4‐PG In Vitro

Studies have demonstrated that interleukin‐6 (IL‐6), a multifunctional proinflammatory cytokine, plays a critical role in wound healing. However, persistent overexpression of IL‐6 is considered a key factor contributing to the progression of fibrosis. IL‐6 promotes the activation of FBs and the expression of α‐SMA by activating the Janus kinase/signal transducer and activator of transcription (JAK/STAT) signaling pathway, thereby driving the fibrotic process (Figure 5g) [35]. Moreover, the secretion of IL‐6 is closely associated with macrophage function, and macrophages are recognized as the primary source of IL‐6 within the fibrotic microenvironment. To further elucidate the role of IL‐6 in fibrosis, this study employed in vitro experiments to analyze the expression levels of IL‐6 markers on macrophages and the amount of IL‐6 secreted. By combining immunofluorescence staining and Western blot (WB) techniques, this study investigated how IL‐6 exacerbates fibrosis by promoting α‐SMA expression in FBs.

First, we evaluated the impact of Tregs on the expression level of IL‐6 and the polarization status of macrophages. Tregs were cocultured with M1 (CD86^+^) macrophages induced by polarization, followed by staining for IL‐6 in the macrophages. The results (Figures S24 and S25) revealed that Tregs were capable of suppressing IL‐6 expression. T cells and M1 (CD86^+^) macrophages were subsequently cocultured in a Transwell system. After the upper layer of T cells was subjected to different treatments, immunofluorescence staining was performed on the macrophages in the lower chamber. Compared with that in the Ctrl group, the fluorescence intensity of IL‐6 in macrophages from the PLGA@LA‐PG, BMP4‐PG, and PLGA@LA‐BMP4‐PG groups was significantly lower. Furthermore, the macrophages in these groups mostly exhibited a rounded morphology, whereas those in the Ctrl group displayed a multipseudopodia morphology (Figure 5h; Figure S26), indicating that the macrophages in the Ctrl group were predominantly polarized into a proinflammatory phenotype with stronger IL‐6 expression. In addition, we analyzed the secretion levels of IL‐6 in macrophages via western blotting (WB) experiments. An analysis of the supernatants of culture media from macrophages subjected to different treatments (Figure 5i; Figure S27) revealed that the levels of IL‐6 secreted by macrophages in the PLGA@LA‐PG, BMP4‐PG, and PLGA@LA‐BMP4‐PG groups were significantly lower than those in the Ctrl group. These findings suggest that LA effectively reduces macrophage IL‐6 secretion through Treg polarization, and combined with the anti‐inflammatory effect of the BMP4 protein, the designed PLGA@LA‐BMP4‐PG bilayer scaffolds had optimal anti‐inflammatory effects.

Furthermore, we assessed the role of IL‐6 in regulating fibrosis. Analysis of α‐SMA expression in FBs stimulated with IL‐6 revealed that α‐SMA expression was markedly higher in IL‐6‐stimulated FBs than in the Ctrl group, indicating that IL‐6 exacerbates fibrosis by promoting FB hyperactivation and differentiation into MFBs (Figure 5j; Figure S28). These experimental results suggest that PLGA@LA‐BMP4‐PG effectively suppresses the release of the profibrotic inflammatory factor IL‐6 from macrophages, thereby mitigating inflammation‐induced fibrosis and reducing scar formation. Therefore, Tregs not only alleviate inflammation‐induced fibrosis but also reduce fibrosis caused by the profibrotic factor TGF‐β, which corresponds to the process of the wound healing inflammatory phase that initiates fibrosis formation and the main phase of fibrogenesis: the proliferative phase. These findings demonstrate that promoting Treg polarization to inhibit fibrosis is a feasible and promising therapeutic strategy.

### 2.7 Effects of the PLGA@LA‐BMP4‐PG Scaffold on Scarless Wound Healing In Vivo

**FIGURE 6:**
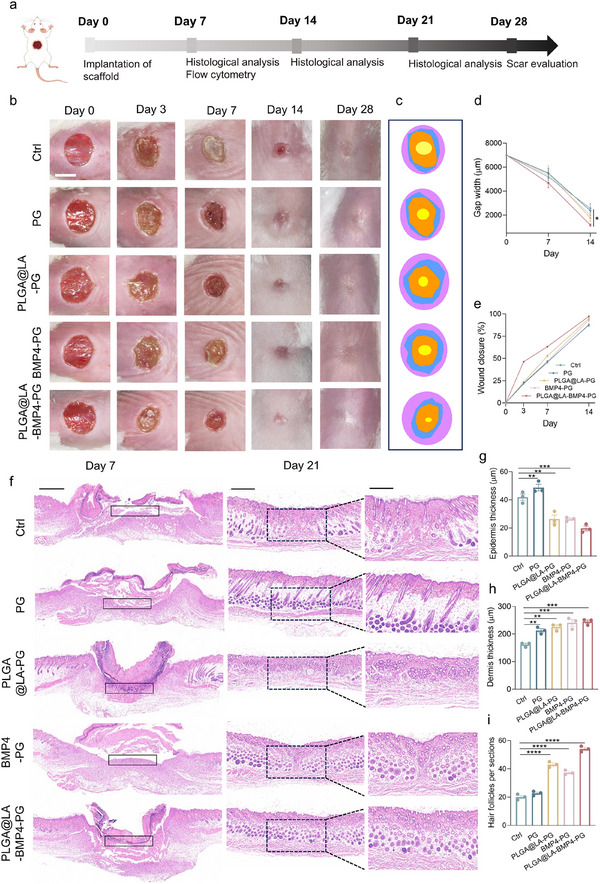
Evaluation of the wound healing process in mice. (a) A workflow for evaluating the wound healing process. (b) Representative digital images of wounds subjected to various treatments at different time points. Scale bar: 5 mm. (c) Traces of wound closure from day 0 to day 14. (d) Records of wound width at different time points (*n* = 5). (e) Wound closure rates at different time points (*n* = 5). (f) Representative images of H&E‐stained wound tissue at days 7 and 21. Scale bar: 1 mm. (g) Epidermal thickness (µm). (h) Dermis thickness (µm). (i) Number of hair follicles at the wound regeneration site. (*n* = 3 biologically independent samples in (g–i)). The data are presented as the means ± SDs. The P values were calculated via one‐way analysis of variance (ANOVA). *P < 0.05, **P < 0.01, ***P < 0.001 and ****P < 0.0001.

Given the promising in vitro results showing that bilayer scaffolds promote cell proliferation and migration, reduce inflammation and fibrosis, and enhance tissue regeneration, we further studied their effects on wound healing in a full‐thickness skin defect model in the dorsal skin of mice. The procedure used to establish the wound model is shown in Figure 6a. After full‐thickness skin defects were created, scaffolds containing different functional components were implanted into the wounds. Histological and immunohistochemical analyses of the wound tissues were performed on days 7, 14, and 21, with continuous photographic records of wound healing throughout the process.

At the macroscopic level, wounds in the groups treated with the functional components LA and BMP4 exhibited accelerated healing across multiple time points compared with those in the Ctrl and PG groups (Figure 6b–e). Compared with the Ctrl group, the PG group demonstrated a faster healing rate, suggesting that the piezoelectric effect generated by mechanical stress from PLA scaffold‐mediated skin tissue stretching enhanced wound healing. Under electric field stimulation, dermal stromal cells exhibit accelerated proliferation and migration, thereby promoting tissue regeneration. Notably, the wounds in the PLGA@LA‐PG and BMP4‐PG groups were significantly faster than those in both the Ctrl and PG groups were. These findings indicate that LA acts as an energy substrate to augment skin cell metabolism and tissue regeneration, whereas BMP4, as a growth factor, facilitates angiogenesis and regulates epithelial cell proliferation. Moreover, the PLGA@LA‐BMP4‐PG group exhibited the most rapid healing kinetics among all the groups, demonstrating synergistic therapeutic effects between the PLGA@LA NPs and BMP4 in accelerating wound repair. By day 28, the tissues in the PLGA@LA‐BMP4‐PG group appeared markedly smoother and flatter than those in the Ctrl group did, with significantly reduced scarring, indicating effective suppression of tissue fibrosis and scar formation through the combined action of PLGA@LA NPs and BMP4.

Histological changes in the wound tissues were assessed via hematoxylin and eosin (H&E) staining and Masson's trichrome staining (Figure 6f–i; Figure S29). H&E staining on day 7 revealed distinct neovascularized granulation tissue formation at the wound centers in the PLGA@LA‐PG, BMP4‐PG, and PLGA@LA‐BMP4‐PG groups, whereas the Ctrl and PG groups presented disorganized, loose fibrous tissue indicative of prolonged inflammatory phase progression (black boxes). These findings demonstrate the anti‐inflammatory and tissue regeneration‐promoting capabilities of LA and BMP4, which is consistent with their observed in vitro effects on immune cell polarization, proliferation, and migration. By day 14, H&E analysis revealed significantly reduced wound dimensions and improved tissue organization in the treatment groups compared with the control groups, with the BMP4‐PG and PLGA@LA‐BMP4‐PG groups displaying particularly smooth tissue surfaces, suggesting direct BMP4‐mediated inhibition of fibrotic processes. On day 21, superior healing quality was evident in the treatment groups through complete epidermal‐dermal layer restoration, increased dermal thickness, and enhanced skin appendage regeneration (hair follicles and sebaceous glands) in the PLGA@LA‐PG and PLGA@LA‐BMP4‐PG groups. This regeneration of functional skin structures represents a critical indicator of high‐quality wound healing. In addition, we found that the situation of neonatal hair follicles in the BMP4‐PG group was also very impressive, probably because although BMP4 puts hair follicle stem cells in the resting phase and delays the activation of hair follicle stem cells, this also maintains the stemness of hair follicle stem cells, which leads to strong differentiation potential in the long term, thus facilitating the regeneration of hair follicles [36].

**FIGURE 7:**
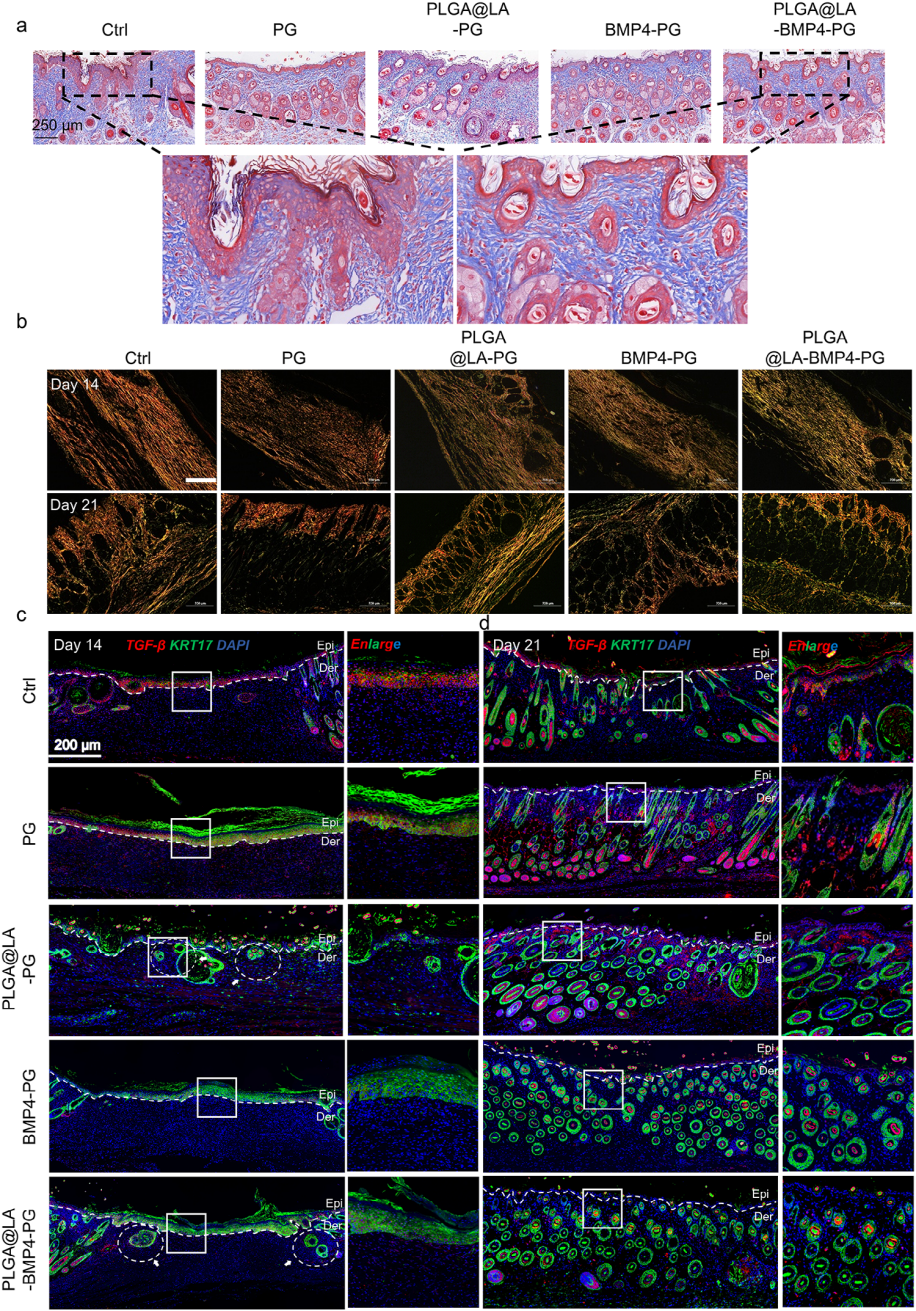
Scarring assessment of dorsal skin tissue in mice. (a) Representative Masson‐stained image of day 21 wound tissue. Scale bar: 250 µm. (b) Representative image of Sirius Red‐stained wounds on days 14 and 21. Scale bar: 200 µm. (c,d) Representative images of TGF‐β and KRT17 fluorescence staining of skin tissue on days 14 and 21. Scale bar: 200 µm.

Masson staining further confirmed these findings (Figure 7a; Figure S30), revealing orderly collagen fiber alignment and reduced deposition in the treatment groups compared with the control groups. The magnified images revealed minimal muscle fiber content and organized collagen architecture in the PLGA@LA‐BMP4‐PG group, closely resembling native skin morphology, whereas the untreated groups exhibited excessive fibrosis and disordered collagen deposition. Sirius Red staining and immunofluorescence analysis of fibrosis markers were conducted to evaluate the antifibrotic effects. Specifically, the PLGA@LA‐BMP4‐PG group presented increased collagen content, well‐aligned fibers, and a more uniform distribution within the dermis, akin to the structure of normal skin. In contrast, the Ctrl and other groups presented loose and disorganized collagen fibers, which suggested that the PLGA@LA‐BMP4‐PG group presented optimal collagen synthesis and assembly. Sirius Red staining indicated that the Ctrl and PG groups were predominantly composed of type I collagen (red), which is characteristic of mature scar tissue (Figure 7b). In contrast, the PLGA@LA‐BMP4‐PG group presented the highest ratio of type III collagen (green), suggesting a regenerative collagen profile with reduced scarring. Sirius Red quantification revealed significantly lower type I/III collagen ratios in the treatment groups than in the Ctrl group, with PLGA@LA‐BMP4‐PG showing the most favorable profile (closest to normal skin) (Figures S31 and S32). Costaining for TGF‐β (fibrosis marker, red) and KRT17 (hair follicle marker, green) on day 14 revealed markedly lower TGF‐β expression in the treatment groups than in the control groups. Notably, nascent hair follicle structures (white dashed circles) were observed in the PLGA@LA‐PG and PLGA@LA‐BMP4‐PG groups, confirming the in vivo efficacy of LA in promoting skin appendage regeneration via Treg polarization (Figure 7c; Figures S33 and S34). By day 21, the PLGA@LA‐BMP4‐PG group presented substantially fewer fibrotic markers and greater hair follicle density than the control group did, confirming the dual functionality of the bilayer scaffold in restoring both structural and functional skin properties (Figure 7d; Figures S35 and S36). In addition, we performed histochemical staining analysis of MFBs in wound skin tissues to visually assess FB activation status. The staining results (Figures S37–S40) demonstrated that the expression levels of MFB markers in the PLGA@LA‐PG, BMP4‐PG, and PLGA@LA‐BMP4‐PG groups were significantly lower than those in the PG and Ctrl groups at days 14 and 21. This observation indicates that during the proliferative phase, LA effectively prevents excessive FB activation through Treg polarization, whereas BMP4 directly suppresses MFB generation by inhibiting FB activation signaling pathways. Notably, we observed greater α‐SMA expression in the PG group than in the Ctrl group on day 21, which may be attributed to the PLA scaffold promoting FB proliferation, thereby enabling more FBs to differentiate into MFBs in response to wound healing signals. Throughout both time points, the PLGA@LA‐BMP4‐PG group exhibited optimal antifibrotic efficacy, demonstrating that the synergistic combination of LA and BMP4 achieves superior antiscarring effects through complementary mechanisms of action.

Comprehensive histological and immunohistochemical analyses confirmed that the bilayer scaffold significantly enhanced epithelialization while inhibiting fibrosis. The synergistic interplay between LA and BMP4 modulates the immune microenvironment and suppresses MFB differentiation, thereby accelerating wound healing with minimal scarring. These findings provide mechanistic insights into the therapeutic potential of the scaffold for functional skin regeneration.

### 2.8 In Vivo Regulation of the Immune Environment of Wounds by PLGA@LA‐BMP4‐PG

To verify whether the bilayer scaffold modulates the in vivo immune environment in a manner consistent with its effects on immune cell polarization in vitro and to explore the relationship between reduced fibrosis and immune environment regulation after treatment, we performed flow cytometry on immune cells in the wound tissue of mice on day 7. We also performed immunofluorescence analysis of FOXP3 and IL‐6 expression levels in the tissue on days 7 and 14.

**FIGURE 8:**
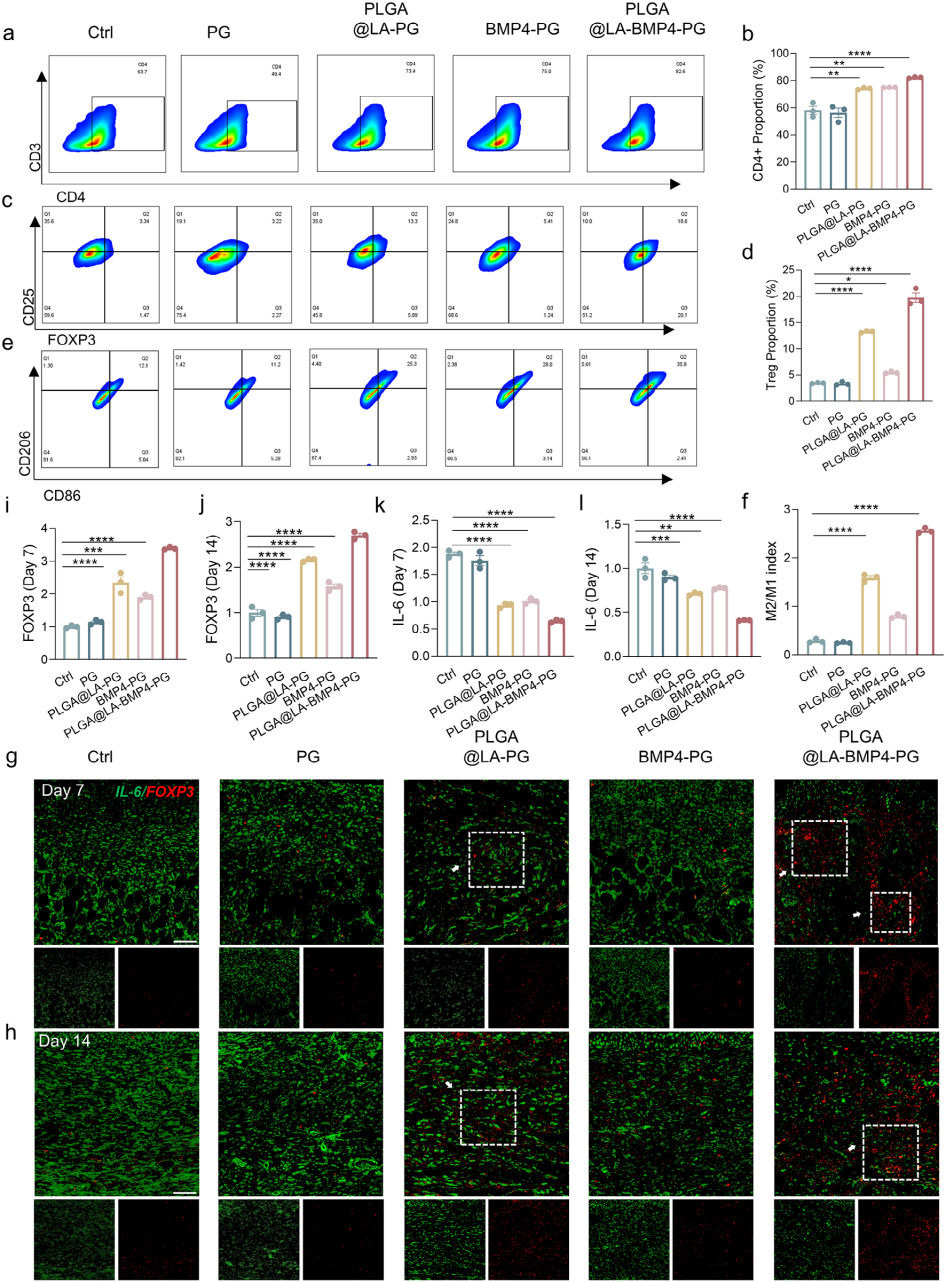
Immunological analysis of wound tissue. (a) Representative flow plots of the results of CD4^+^ T‐cell polarization in the wound tissue. (b) Quantification of the results of CD4^+^ T‐cell polarization (*n* = 3). (c) Representative plots of the flow analysis results of Treg polarization in the wound tissue. (d) Quantification of Treg polarization (*n* = 3). (e) Representative plots of the results of flow cytometry analysis of macrophage polarization in the wound tissue. (f) Quantification of the results of macrophage polarization (*n* = 3). (g,h) Representative fluorescence images of FOXP3 (red) and IL‐6 (green) in the wound tissue on days 7 and 14. (i–l) Quantitative analysis of the fluorescence intensity of FOXP3 and IL‐6 expression (*n* = 3). The data are presented as the means ± SDs. The P values were calculated via one‐way analysis of variance (ANOVA). *P < 0.05, **P < 0.01, ***P < 0.001, and ****P < 0.0001.

Flow cytometric analysis of T cells in day 7 wound tissues (Figure 8a–d; Figures S41–S43) revealed that the BMP4‐PG, PLGA@LA‐PG and PLGA@LA‐BMP4‐PG groups presented greater proportions of CD4^+^ T cells than did the Ctrl group. Compared with the other groups, the PLGA@LA‐PG and PLGA@LA‐BMP4‐PG groups presented fewer CD8^+^ T cells. The observed increase in CD4^+^ T‐cell populations and concomitant reduction in CD8^+^ T‐cell populations in the experimental group compared with those in the control group suggest that the therapeutic agent promotes an immunomodulatory microenvironment conducive to wound repair. CD4^+^ T cells are critical for suppressing excessive inflammation and facilitating tissue regeneration through anticytokine signaling and direct interactions with stromal cells [37]. Conversely, CD8^+^ T cells, as cytotoxic effectors, may exacerbate tissue damage by secreting proinflammatory mediators (e.g., IFN‐γ and TNF‐α) and targeting stressed cells during acute inflammation [38]. Subsequent statistical analysis of Treg ratios within CD4^+^ T‐cell populations revealed significantly elevated Treg percentages in the PLGA@LA‐PG and PLGA@LA‐BMP4‐PG groups relative to those in the other experimental groups. Combined with FOXP3 immunohistochemical staining of Tregs in wound tissues on days 7 and 14 (Figure 8g–j), these results demonstrated a trend consistent with the flow cytometry findings, showing markedly stronger expression of Treg markers in the PLGA@LA‐PG and PLGA@LA‐BMP4‐PG groups than in both the Ctrl and PG groups. These observations align with the in vitro polarization effects of LA on T cells, suggesting that LA promotes Treg polarization in vivo.

Interestingly, we noted that, compared with the control group, the BMP4‐PG group presented increased proportions of Tregs according to both flow cytometry and immunostaining analyses. We hypothesize that this phenomenon may stem from the effects of macrophage polarization on Treg differentiation during immune responses. Flow cytometric evaluation of wounded macrophages on day 7 (Figure 8e,f; Figure S44) revealed significantly higher M2/M1 ratios in the BMP4‐PG, PLGA@LA‐PG, and PLGA@LA‐BMP4‐PG groups than in the control group. These findings suggest that BMP4 may influence Treg polarization through M2 macrophage induction, as M2 macrophages secrete cytokines such as TGF‐β and IL‐10 that regulate T‐cell differentiation. Specifically, TGF‐β directly promotes the differentiation of CD4^+^ T cells into Tregs, while IL‐10 maintains Treg stability through anti‐inflammatory microenvironment modulation. Conversely, proinflammatory cytokines (IL‐6 and TNF‐α) secreted by M1 macrophages inhibit Treg differentiation while promoting Th17/Th1 effector cell polarization. To investigate whether BMP4 influences the differentiation of Tregs by modulating macrophages, we established an in vitro coculture system containing M0 macrophages and T cells. In this system, macrophages in the upper chamber were stimulated with BMP4, and the expression of key Treg markers in the lower chamber was analyzed via flow cytometry. The results demonstrated that BMP4 significantly promoted the polarization of Tregs by inducing phenotypic switching in macrophages (Figures S45 and S46). This mechanism may form a positive feedback loop that suppresses inflammatory responses in vivo, thereby contributing to the maintenance of a sustained anti‐inflammatory immune environment. Additionally, LA released from PLA scaffold degradation in the bilayer system may further contribute to Treg polarization.

Collectively, our in vivo findings demonstrate that the PLGA@LA‐BMP4‐PG scaffolds promote Treg polarization through two mechanisms: direct LA‐mediated effects and BMP4‐induced M2 macrophage polarization, establishing a positive feedback loop. Immunostaining analysis of IL‐6 expression (Figure 8g,h,k,l) revealed significantly lower levels in the treated groups than in the control groups at both days 7 and 14, with more pronounced differences observed on day 7. This temporal pattern likely reflects earlier transitions from the inflammatory phase in the treatment groups to persistent inflammation in the control groups. A comprehensive analysis of immune cell dynamics demonstrated that the PLGA@LA‐BMP4‐PG bilayer scaffold effectively modulates immune cell polarization and intercellular crosstalk to establish a pro‐healing microenvironment. The sustained immunomodulatory effects observed throughout the wound healing stages highlight its clinical potential for therapeutic translation.

### 2.9 Transcriptome Profiles of BMP4‐ and LA‐Mediated Regulation of Wound Healing

**FIGURE 9:**
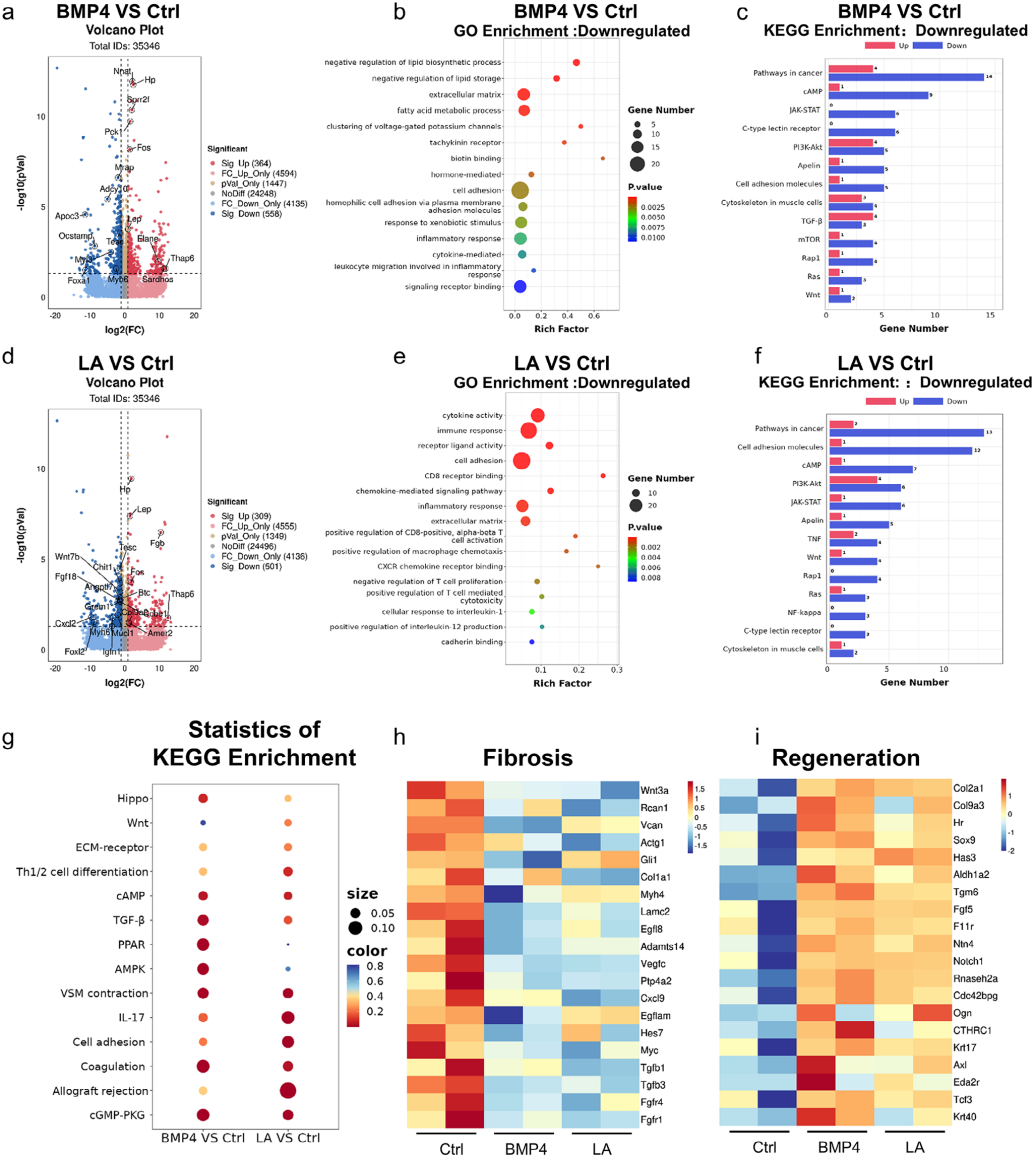
Transcriptome profiles of the BMP4‐ and LA‐mediated regulation of wound healing. (a) Volcano plot of the BMP4 and Ctrl groups. (b,c) GO terms and KEGG pathways enriched in downregulated genes in the BMP4 VS Ctrl group. (d) Volcano plot of the LA group vs the Ctrl group. (e,f) Downregulated enriched GO terms and KEGG pathways in the LA vs Ctrl group. (g) Statistics of KEGG enrichment. (h) Heatmap of genes involved in fibrosis. (i) Heatmap of gene expression involved in gene generation. (*n* = 3 biologically independent samples in (a–i)). The data are presented as the means ± SDs. The P values were calculated via one‐way analysis of variance (ANOVA). *P < 0.05, **P < 0.01, ***P < 0.001, and ****P < 0.0001.

To further elucidate the mechanisms involved in the regulation of scarless healing by the two functional components, LA and BMP4, used in this study, we performed transcriptomic analysis on the skin tissues of mice treated for 28 days (BMP4 group and LA group). Compared with the Ctrl group, the BMP4‐PG group presented extensive differential gene expression (GDE), with a volcano plot showing 4958 upregulated genes and 4693 downregulated genes, among which 364 genes were significantly upregulated and 558 genes were significantly downregulated (GSDE) (Figure 9a). Similarly, the PLGA@LA‐PG group also presented widespread GDE, with a volcano plot revealing 4864 upregulated genes and 4637 downregulated genes, of which 309 were significantly upregulated and 501 were significantly downregulated (GSDE) (Figure 9d). Among these genes, 108 significantly upregulated genes and 185 significantly downregulated genes were common between the BMP4‐PG and PLGA@LA‐PG groups (Figure S47).

We then collected the GSDEs and conducted Gene Ontology (GO) database analysis to describe the properties of the genes and their products, including their molecular functions (MFs), cellular components (CCs), and biological processes (BPs). The results revealed significant enrichment of DEGs in GO terms related to cell adhesion, the immune response, keratinization, cytokine activity, and cell differentiation (Figure S48). We found that GO terms related to fibrosis (e.g., extracellular matrix, cell adhesion, homophilic cell adhesion via plasma membrane adhesion molecules) and the inflammatory immune response (e.g., the inflammatory response and leukocyte migration involved in the inflammatory response) were significantly downregulated in the BMP4‐PG group, indicating that BMP4 can significantly reduce wound inflammation and fibrosis, thereby alleviating scar formation (Figure 9b). Additionally, GO terms associated with cell proliferation and tissue component regeneration (e.g., germline stem cell division, regulation of vascular endothelial cell proliferation, intermediate filament organization) were significantly enriched (Figure S49), suggesting that BMP4 also plays a role in promoting tissue structure regeneration. In the PLGA@LA‐PG group, GO terms related to fibrosis (e.g., extracellular matrix, cell adhesion, and cadherin binding) and the inflammatory response (e.g., the inflammatory response, positive regulation of macrophage chemotaxis, the cellular response to IL‐1, and positive regulation of IL‐12 production) were significantly downregulated (Figure 9e), indicating that LA effectively reduces extracellular matrix production and cell adhesion to inhibit scarring while also reducing inflammation by inhibiting macrophage polarization and decreasing the secretion of proinflammatory cytokines (IL‐1 and IL‐12), which are crucial for mitigating fibrosis. Notably, GO terms related to the CD8 receptor and α‐β T cells were downregulated in the PLGA@LA‐PG group. CD8^+^ T cells are cytotoxic cells, and during immune responses, Tregs typically suppress the activity of CD8^+^ T cells to prevent the immune system from attacking normal tissue. α‐β T cells, which include both CD4^+^ and CD8^+^ T cells, are effector immune cells responsible for antigen recognition and immune activation. Furthermore, the balance between α‐β T cells and Tregs is crucial for immune defense [39]. Thus, the downregulation of biological processes and molecular functions related to CD8^+^ T cells and α‐β T cells may be due to the promotion of Treg polarization by LA, which is consistent with our previous findings. Additionally, GO terms related to skin epidermal regeneration (keratinization, structural constituent of the skin epidermis), cell proliferation (germline stem cells), and inhibition of fibrosis (fibrinolysis) were significantly enriched in the PLGA@LA‐PG group (Figure S50), further indicating that LA can suppress skin fibrosis.

Furthermore, we performed Kyoto Encyclopedia of Genes and Genomes (KEGG) pathway enrichment analysis on DEGs between the BMP4 and LA groups and the Ctrl group. The results revealed significant enrichment of GDEs in typical fibrosis‐associated pathways, including ECM‐receptor interaction, Wnt (Wingless/Integrated), TGF‐β (Transforming Growth Factor‐beta), and cell adhesion molecules (Figure 9g). Subsequent quantification of downregulated genes in corresponding pathways revealed marked suppression of fibrotic pathways, particularly those related to cell adhesion molecules, the TGF‐β signaling pathway, and the Wnt signaling pathway, in the BMP4 group (Figure 9c). These findings confirm that BMP4 attenuates scar formation through direct inhibition of fibrotic signaling pathways. Similarly, the LA group exhibited downregulation of fibrosis‐related pathways, including cell adhesion molecules, the Janus tyrosine kinase‐signal transducer and activator of transcription (JAK‐STAT) signaling pathway, and the Wnt signaling pathway (Figure 9f). Concurrently, the inflammatory pathway mediated by TNF (tumor necrosis factor) was significantly suppressed. These findings indicate that LA effectively and persistently inhibits wound inflammation while reducing FB activation and cellular adhesion. The dual mechanisms involving immunomodulatory effects and suppression of critical signaling pathways collectively mitigate fibrotic progression, demonstrating the potential of LA for achieving scarless wound healing.

Next, we compared the expression levels of genes related to fibrosis and regeneration among the genes significantly differentially expressed between the groups. Heatmap analysis revealed that genes related to fibrosis (e.g., Wnt3a, Actg1, Col1a1, Myc, Tgfb1, and Fgr1) presented significantly greater expression in the Ctrl group than in the BMP4‐PG and PLGA@LA‐PG groups did, whereas genes related to skin tissue (e.g., Col2a1 and Col9a3) and skin appendage (e.g., Sox9, Notch1, Krt17, and Krt40) regeneration presented significantly lower expression in the Ctrl group than in the BMP4‐PG and PLGA@LA‐PG groups did. These findings suggest that both BMP4 and LA can effectively promote the complete regeneration of defective skin tissue, including structural and functional restoration, greatly improving healing outcomes.

### 2.10 Scarless Wound Healing Regulated by PLGA@LA‐BMP4‐PG in a Rabbit Ear Wound Model

**FIGURE 10:**
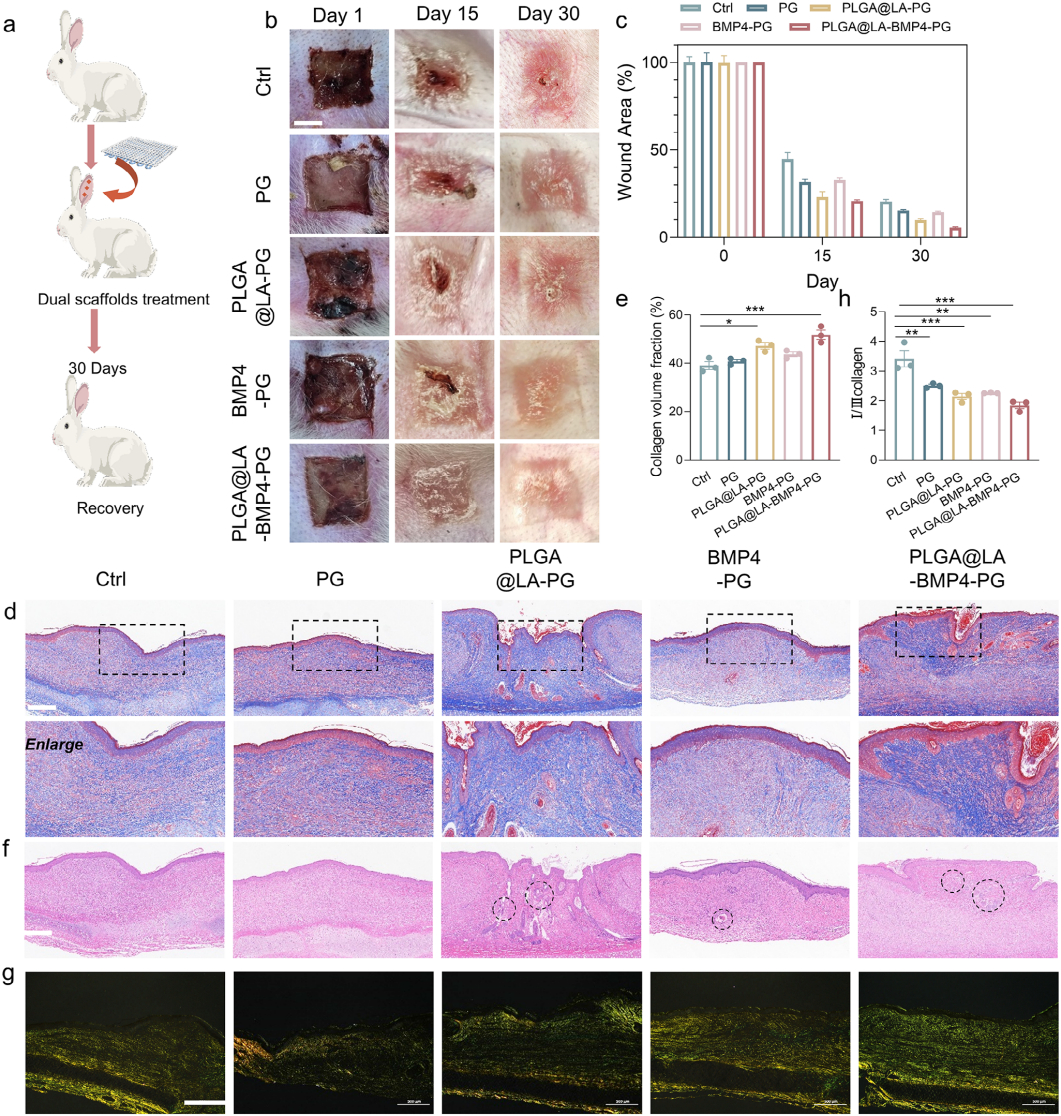
Scarless wound healing regulated by PLGA@LA‐BMP4‐PG in rabbits. (a) Schematic diagram of the experimental procedure. (b) Representative digital images of wounds subjected to various treatments at different time points. Scale bar: 5 mm. (c) Wound area at different time points (*n* = 3). (d) Representative images of Masson staining of wound tissue on day 30. The black box enlargement shows the wound center area. Scale bar: 1 mm. (e) Quantification of the collagen volume fraction (*n* = 3). (f) Representative images of H&E‐stained wound tissue on day 30. Scale bar: 1 mm. (g) Representative image of Sirius Red‐stained wounds on day 30. Scale bar: 500 µm. (h) Quantification of I/III collagen (*n* = 3). The data are presented as the means ± SDs. The P values were calculated via one‐way analysis of variance (ANOVA). *P < 0.05, **P < 0.01, ***P < 0.001 and ****P < 0.0001.

To visually assess the ability of the bilayer scaffold to reduce scar formation, wounds on the rabbit ear were treated, and both the healing rate during the recovery process and the severity of scarring after 30 days were observed (Figure 10a). Macroscopically, the healing rate of the treatment group was significantly faster than that of the Ctrl group. As shown in Figure 10b, during the wound healing process in rabbit ears, wounds in the PLGA@LA‐BMP4‐PG group were almost completely closed by day 15, whereas those in the Ctrl group had not fully healed, and the other treatment groups still exhibited scab formation. By day 30, the scars in the treatment group were noticeably less severe than those in the Ctrl group were, with the treated scars exhibiting a color closer to that of normal skin. Specifically, the newly formed tissue in the PLGA@LA‐BMP4‐PG group presented the lightest color and most closely resembled uninjured skin, whereas the Ctrl and PG groups presented a darker pink hue, suggesting more severe scarring. In addition, the newly formed skin tissue in the Ctrl group appeared rougher and more wrinkled, whereas the regenerated tissue in the treated group was smoother and had a more even surface. These macroscopic observations provide direct evidence that the bilayer scaffold could effectively reduce scar severity and contribute to the restoration of skin aesthetics, highlighting its potential application in scarless healing (Figure 10b,c).

Additionally, tissue samples from the rabbit ears were collected on day 30 for histological analysis. Masson's trichrome staining (Figure 10d,e) revealed that, compared with the Ctrl group, all the treatment groups presented increased deposition of newly formed collagen fibers along with reduced MFB content. In particular, the PLGA@LA‐BMP4‐PG group presented the most abundant and well‐aligned collagen fibers in the wound center, indicating that enhanced collagen regeneration was promoted by the bilayer scaffold. Notably, the collagen content in both the BMP4‐PG and PLGA@LA‐PG groups was also greater than that in the Ctrl group, suggesting that LA mitigates inflammatory responses by promoting Treg polarization, thereby fostering a favorable immune microenvironment for collagen synthesis. Moreover, BMP4 inhibited the transformation of FBs into MFBs, reducing excessive ECM deposition and alleviating fibrosis. The synergistic action of LA and BMP4 in immunomodulation and cell fate regulation significantly attenuated scar formation. Furthermore, the H&E staining results (Figure 10f) indicated that the regenerated skin in the PLGA@LA‐BMP4‐PG group contained more skin appendages, such as hair follicles (indicated by black dashed lines). The PLGA@LA‐PG and BMP4‐PG groups also exhibited a certain degree of hair follicle regeneration, which is consistent with previous findings from cell experiments: LA facilitates the proliferation and differentiation of hair follicle stem cells via Treg activation (Figure S19), whereas BMP4 supports a regenerative microenvironment through inflammation modulation (Figures S45 and S46). Together, they promote not only structural restoration but also functional recovery of the skin, addressing a critical need in regenerative wound therapy.

To further evaluate the collagen composition, Sirius Red staining was performed under polarized light (Figure 10g,h), where type I collagen appears red/orange and type III collagen appears green. Compared with that in the Ctrl group, the regenerated tissue in the PLGA@LA‐BMP4‐PG group contained a significantly greater proportion of type III collagen fibers, along with better collagen alignment. This pattern suggests a regenerative collagen phenotype of wound healing, confirming from both morphological and biochemical perspectives that the scaffold significantly improved healing quality. Collectively, these histological findings provide robust evidence that the scaffold reduces scar severity through coordinated mechanisms involving immunomodulation, collagen remodeling, and skin appendage regeneration.

The PLGA@LA‐BMP4‐PG scaffold promotes scarless wound healing through multiple synergistic mechanisms: LA induces Treg polarization, effectively suppressing excessive inflammation and optimizing the immune microenvironment; BMP4 inhibits the transdifferentiation of FBs into MFBs, thereby reducing the degree of fibrosis; furthermore, the combined action of LA and BMP4 activates the regeneration of skin appendages such as hair follicles, achieving true structural regeneration and supporting the restoration of skin functionality. Ultimately, this multipathway synergistic regulation enables comprehensive scarless healing.

## 3 Conclusions

This study introduces a novel and promising bilayer scaffold that combines PLA and GelMA and is designed to enhance wound healing and reduce scar formation. By incorporating functional substances such as LA and BMP4, the scaffold not only accelerates tissue regeneration but also addresses the challenges of fibrosis, a key factor in scar formation. The innovative design of the scaffold—featuring a piezoelectric PLA upper layer and a drug‐delivery GelMA lower layer—enables dynamic interactions with the wound microenvironment, promoting cellular proliferation, migration, and functional tissue regeneration. LA plays a pivotal role in modulating the immune environment by promoting Treg polarization and modulating the macrophage phenotype, which facilitates the resolution of inflammation and prevents excessive fibrosis. BMP4, on the other hand, inhibits MFB activation by modulating the TGF‐β/Smad signaling pathway, thereby reducing excessive extracellular matrix deposition and thus minimizing scar tissue formation.

The in vitro findings confirmed the ability of the scaffold to induce the polarization of Tregs, promote the M2 macrophage phenotype, and regulate FB behavior, all of which contribute to a more favorable environment for wound healing. Moreover, the in vivo results support these findings, demonstrating that the bilayer scaffold accelerates wound closure, reduces inflammation, and significantly enhances tissue regeneration while preventing excessive scar formation. The synergistic action of LA and BMP4 in this system ensures that both the inflammatory and proliferative phases of wound healing are effectively controlled, leading to more organized and functional tissue repair.

The ability of the PLA scaffold to generate an electric field further contributes to its therapeutic effects by stimulating cell migration and enhancing the cellular functions essential for wound closure. The mechanical properties of the scaffold, including its flexibility and tensile strength, ensure that it can withstand the stresses of normal movement, making it a suitable candidate for practical use in clinical settings.

In conclusion, the proposed multifunctional scaffold has significant clinical advantages over existing antiscar therapies, such as hyaluronic acid dressings, silicone gels, and TGF‐β pathway inhibitors. Current treatments often focus on a single aspect of wound management, such as moisture provision or isolated biochemical modulation. Our strategy integrates piezoelectric stimulation, localized immunomodulation, and targeted antifibrotic activity into a unified platform. This combination not only supports faster and more organized tissue repair but also actively prevents pathological scarring through multilevel regulation of the wound healing process. Moreover, this scaffold has strong potential for application in challenging clinical scenarios, including chronic wounds, refractory ulcers, and postsurgical scar prevention, where current therapies show limited efficacy. Its ability to promote a regenerative immune microenvironment, coupled with tunable release kinetics and biomimetic physical properties, underscores its high translational value.

However, several key challenges regarding clinical translation must be proactively addressed. The complex pathological microenvironment of chronic wounds is characterized by persistent inflammation and microbial colonization leading to biofilm formation, and impaired cellular responses pose a significant barrier to consistent scaffold performance. This environment may alter drug release kinetics, impede cellular integration, and ultimately compromise therapeutic efficacy, necessitating evaluation under clinically relevant disease conditions. Additionally, interindividual variability in immune regulation presents another layer of complexity. Divergent healing outcomes across patients, influenced by factors such as age, comorbidities, and genetic background, underscore the potential need for patient‐specific dosing regimens or customizable scaffold configurations to ensure broad applicability. Furthermore, while the use of biomimetic materials minimizes the risk, the potential for immune rejection of the delivery system cannot be entirely discounted. A thorough assessment of immunogenicity in human‐relevant models is therefore imperative to validate the safety profile and long‐term biocompatibility of the scaffold. The complexity of production, the long‐term stability of the scaffold in vivo, and potential variations in response depending on individual patient conditions must be further explored. Additionally, although the combination of LA and BMP4 resulted in superior wound healing outcomes, the precise dosage and release kinetics of these agents must be optimized to avoid potential side effects such as excessive acidity or uncontrolled inflammation.

Addressing these challenges necessitates the development of smarter, adaptive systems. Future designs should pivot toward closed‐loop feedback mechanisms that release therapeutics in response to specific wound biomarkers (e.g., pH and MMPs), thereby autonomously adapting to the wound status. The combination of advanced strategies to dynamically modulate immune cell behavior and achieve precise regulation of the immune microenvironment could pave the way for personalized and precision wound care. Furthermore, the potential of this scaffold to promote the regeneration of skin appendages, such as hair follicles, which are often lost in scar tissue, should be explored more deeply. Understanding the mechanisms through which this scaffold influences skin regeneration and immune modulation could provide new insights into tissue engineering and regenerative medicine.

In summary, the bilayer scaffold presented in this study represents a novel approach for improving wound healing and reducing scar formation. Its ability to modulate the immune response, combined with its mechanical and biological properties, offers a promising strategy for the treatment of chronic wounds and the prevention of scarring, paving the way for future therapeutic applications in tissue regeneration.

## 4 Experimental Section

### 4.1 Ethical Approval and Informed Consent

The animal experiments conducted in this study were approved by the Animal Ethics Committee of Tianjin University Laboratory Animal Center and were carried out in strict accordance with the Guidelines for the Care and Use of Laboratory Animals of Tianjin University. The experimental protocols were specifically approved by the Animal Ethics Committee of the Tianjin University Laboratory Animal Center (Tianjin, China) under Approval No. TJUE‐2024‐349. The human experiments conducted in this study were approved by the Scientific Research Ethics Committee of Tianjin University under Approval No. TJUE2025‐H‐X‐019. The participants who participated in the human experiment signed informed consent forms.

### 4.2 In Vivo Wound Healing Study in Mice

Female BALB/C mice weighing approximately 20 g (4–5 weeks) were obtained from Spafford. The mice were randomly divided into 5 groups: (1) PBS; (2) PG; (3) BMP4‐PG; (4) PLGA@LA‐PG; and (5) PLGA@LA‐BMP4‐PG. A total wound was made on the back using a disposable perforator with a diameter of 7 mm to a depth of 2 mm. Postoperative application was performed on the wound, which was renewed every two days. The healing process was monitored by a digital camera, and the wound area was quantified via ImageJ software.

### 4.3 In Vivo Flow Cytometry Analysis

On day 7, three mice per group were randomly selected for euthanasia, and tissues were collected from the wounds of the mice. The tissues were lysed with 1 µg/mL of collagen type I enzyme, collagen type IV enzyme and 0.3 µg/mL DNase for 1 h at 37 °C, and the lysed tissues were collected and milled with sieve mesh for the preparation of a cell suspension, which was then treated with anti‐CD3, anti‐CD4, anti‐CD8a, anti‐CD25, anti‐FOXP3, anti‐CD11b, anti‐CD86, and anti‐CD206 antibodies, after which the tissues were analyzed for T cells and macrophages via flow cytometry.

### 4.4 Histopathology and Immunofluorescence Microscopy of the Mice

On days 7, 14, and 21, the animals were euthanized, and wound tissues were collected. The tissues were then fixed in 10% formalin neutral buffer solution (Sigma‒Aldrich), embedded in paraffin and stained with H&E (days 7, 14, and 21)/Masson (day 21)/Sirius Red (days 14 and 21) according to the manufacturer's manual. Histological images were obtained under 3DHISTECH. The thickness and scar area were quantified via NDP View 2 software. The collagen volume fraction was quantified via ImageJ software (v1.53t). On days 7 and 14, three mice per group were randomly selected for euthanasia, and the wound tissues were collected, paraffin‐embedded and then sectioned. Anti‐FOXP3 and anti‐IL‐6 were incubated with the sections overnight for staining, and then, the corresponding 2 antisera were incubated with the sections for 1 h. Finally, the samples were stained with DAPI and scanned for imaging by 3DHISTECH. The fluorescence intensity was quantified via ImageJ software (v1.53t). On days 14 and 21, three mice per group were randomly selected for euthanasia, and the wound tissues were collected, paraffin‐embedded and sectioned. The sections were incubated with anti‐TGF‐β, anti‐KRT17 and anti‐α‐SMA antibodies overnight for staining, and then, the corresponding 2 antisera were incubated with the sections for 1 h, stained with DAPI and scanned for imaging by 3DHISTECH. The fluorescence intensity was quantified via ImageJ software (v1.53t).

### 4.5 Transcriptomic Analysis

The mice used in the experiment were 4‐week‐old female mice. On the 21st day post‐wounding, skin tissue was collected from the wound area. After being washed with PBS (phosphate‐buffered saline) and the superficial fat and impurities were removed, the tissue was immediately frozen in liquid nitrogen and stored at −80°C until subsequent RNA extraction. Total RNA was extracted from the skin tissue via TRIzol reagent (Invitrogen) according to the manufacturer's instructions. Specifically, the frozen skin tissue was ground into powder in liquid nitrogen, followed by the addition of TRIzol reagent. The tissue was then subjected to lysis, phase separation, and centrifugation, resulting in the extraction of total RNA from the supernatant. The RNA concentration and purity were assessed via a NanoDrop spectrophotometer, and RNA quality was evaluated via a Bioanalyzer, ensuring an RNA integrity number (RIN) greater than 7.0. Additionally, GO enrichment analysis and KEGG pathway enrichment analysis were performed via the OmicStudio tool.

### 4.6 In Vivo Wound Healing Study in Rabbits

Female New Zealand White rabbits weighing approximately 2–3 kg (3–4 months of age) were used. The rabbits were randomly divided into 5 groups: (1) PBS; (2) PG; (3) BMP4‐PG; (4) PLGA@LA‐PG; and (5) PLGA@LA‐BMP4‐PG. Full‐thickness skin that was 1 square centimeter in size at the abdomen of the ear was collected, and a postoperative application was made to the wound. The healing process was monitored by a digital camera, and the wound area was quantified via ImageJ software.

### 4.7 Histopathology and Immunofluorescence Microscopy in Rabbits

On day 30, one rabbit from each group was randomly selected, and full‐thickness skin tissue from the wound healing site on the aural abdomen was collected for tissue section analysis. The tissues were then fixed in 10% formalin neutral buffer solution (Sigma‒Aldrich), embedded in paraffin and subjected to H&E/Masson/Sirius Red staining according to the manufacturer's manual. Histological images were obtained under 3DHISTECH. The thickness and scar area were quantified via NDP View 2 software. The collagen volume fraction was quantified via ImageJ software (v1.53t).

### 4.8 Statistical Analysis

The experiments described above were repeated three times, and the sample sizes are presented in the figures and tables. All the data are presented as the means ± standard deviations (SDs). Data preprocessing included standardization to eliminate variability and ensure comparability across different experiments. A two‐tailed unpaired t test was used to assess statistical significance between two groups, whereas comparisons among more than three groups were performed via one‐way analysis of variance (ANOVA). Statistical analysis was conducted with GraphPad Prism 9 software. Statistical significance is indicated as *P < 0.05, **P < 0.01, and ***P < 0.001, with *P* < 0.05 considered statistically significant. Flow cytometry data were analyzed via FlowJo v10.8.1 software, and fluorescence microscopy (FL) images were processed with ImageJ (version 1.8.0).

## Author Contributions

Y.Y. and M.K. contributed equally to this work. X.J. conceptualized and designed the study. Y.Y. and M.K. performed the experiments and drafted the manuscript. J.S. and R.L. contributed to data collection, analysis, and interpretation. Y.Z. and X.L. validated the experimental results and helped with data visualization. R.L. and Y.K. contributed to software development and data validation. S.Z., B.Y., and X.J. oversaw the project, secured funding, and reviewed and edited the manuscript for final approval.

## Conflicts of Interest

The authors declare no conflict of interest.

## Supporting information




**Supporting file 1**: advs74391‐sup‐0001‐SuppMat.docx

## Data Availability

The data that support the findings of this study are available from the corresponding author upon reasonable request.
