# Supplementary material for: Promoting Treg Polarization‐Mediated Anti‐Scar and Appendage Regeneration in Wound Healing
Source: Adv Sci (Weinh). 2026 Feb 24;13(23):e10815. doi: 10.1002/advs.202510815 (PMC13104105; doi:10.1002/advs.202510815)
Supplement: Supplementary file 1 — Supporting file 1: advs74391‐sup‐0001‐SuppMat.docx [file ADVS-13-e10815-s001.docx]

**Supporting Information**

**Promoting** **Treg Polarization-Mediated Anti-Scar and Appendage Regeneration in Wound Healing**

Yiwen Yang, Mingjie Kuang*, Jiacheng Shi, Ruiyan Li, Yuhan Zhang, Xinting Liu, Runtan Li, Yong Kang, Bin Yao*, Shuquan Zhang*, Xiaoyuan Ji*

Y. Yang, J. Shi, R. Li, Y. Zhang, X. Liu, R. Li, Y. Kang, B. Yao, X. Ji

Academy of Medical Engineering and Translational Medicine, Medical College, Tianjin University, Tianjin 300072, China.

R. Li, Y. Zhang, Y. Kang, B. Yao, X. Ji

State Key Laboratory of Advanced Medical Materials and Devices, Tianjin University, Tianjin 300072, China.

M. Kuang

Department of Orthopedics, Shandong Provincial Hospital Affiliated with Shandong First Medical University, Jinan, Shandong 250021, China.

S. Zhang

Integrated Chinese and Western Medicine Hospital, Tianjin University, Tianjin 300100, China

X. Ji

Department of Gastric Surgery, Tianjin Medical University Cancer Institute & Hospital, National Clinical Research Center for Cancer; Tianjin Key Laboratory of Digestive Cancer; Tianjin’s Clinical Research Center for Cancer, Tianjin 300060, China.

*Corresponding author. Email: [jixiaoyuan@tju.edu.cn](mailto:jixiaoyuan@tju.edu.cn) (X. J.), [zhangshuquan666@126.com](mailto:zhangshuquan666@126.com) (S. Z.), [hongyaobin_1212@tju.edu.cn](mailto:hongyaobin_1212@tju.edu.cn) (B. Y.), [doctorkmj@tmu.edu.cn](mailto:doctorkmj@tmu.edu.cn) (M.K.)

*Materials*: PLA (Mw:8000, CAS:26100-51-6) was purchased from Macklin. Gelatin from porcine skin (CAS:9000-70-8) was purchased from Vetec. PLGA (LA/GA = 50:50, Mw = 88--117, Jinan Daigang Biomaterial Co. Ltd.) L-lactic acid (LA) (Lot. No. 100+L02000NCL1) purchased from HEOWNS. PVA (Mw: 13000--23000, CAS: 9002--89--5) was purchased from Sigma‒Aldrich. Recombinant bone morphogenetic protein 4 (BMP4) was purchased from Cloud Clone Corp. Dichloromethane (Mw:84.93, Lot. No. D929386C3) purchased from Shanghai Acmec Biochemical Co., Ltd. CD45, CD11b, CD86, CD206, CD3, CD4, CD8a, CD25, and Foxp3 antibodies for FACS analysis were obtained from Biolegend, Inc. A Cell Counting Kit-8 (CCK8) was purchased from Solarbio. Calcein-AM/propidium iodide (PI) was purchased from Beyotime Biotechnology. DMEM basic (1×) and PBS were purchased from Gibco. Anti-α-SMA (GB111364-100)/TGF-β (GB11179-100)/KRT17 (GB11363-100)/FOXP3 (GB112325-100)/IL-6 (GB11117-100) were purchased from Servicebio.

*In vitro analysis of the effect of Tregs on fibrosis*: Fibroblasts were seeded in Petri dishes and cultured at 37 °C with 5% CO₂ for 24 h. Subsequently, 1 µg/mL LPS was added to all the cells. The cells were then subjected to the following treatments: (1) PBS; (2) coculture with T cells in a Transwell system, where T cells were placed in the upper chamber and where fibroblasts were placed in the lower chamber; and (3) coculture with Tregs in a Transwell system, with Tregs in the upper chamber and fibroblasts in the lower chamber. After 2 days of treatment, the cells were stained with DAPI, anti-TGF-β, and anti-α-SMA antibodies. Fluorescence images of different channels, as well as merged images, were captured via a Leica confocal microscope.

*In vitro flow cytometry analysis of the effects of LA on T cells and macrophages*: T cells were extracted from the spleens of mice at approximately 4 weeks of age, spread into 6-well plates and cultured for 1 day. The T cells were divided into two groups. The control group was left untreated, and the other group was treated with 10 mM LA. After 3 days of culture, the cells were collected and stained with anti-CD3 FITC, anti-CD4 APC, anti-CD25 PE/Cy7 and anti-Foxp3 BV421 antibodies, followed by flow cytometry analysis of the proportion of each subpopulation. Single cells were first gated on the basis of light scatter properties under appropriately adjusted voltage settings. From the single-cell gate, CD3^+^ T cells were identified on the basis of FITC positivity. Subsequently, CD4^+^ T cells were selected from the CD3^+^ T-cell population by APC-positive expression. Finally, Tregs within the CD4^+^ T-cell gate were defined as those that were double positive for PC7 and PB450.

RAW 264.7 cells were treated with 1 µg/mL LPS for 24 h and stimulated by the addition of 10 mM LA for 3 days. Controls were left untreated, and the cells were collected and stained with anti-CD11b BV605, anti-CD86 BV650, and anti-CD206 PE/610 antibodies and then analyzed via a flow assay to determine the M1/M2 macrophage/M1/M2 macrophage ratio. Single cells were initially gated on the basis of light scatter properties via appropriately adjusted voltage settings. From the single-cell gate, CD11b^+^ macrophages were identified by positive staining for Violet 610. Within the CD11b^+^ macrophage population, M1 macrophages were subsequently defined as Violet 660-positive (CD86^+^), whereas M2 macrophages were selected on the basis of ECD positivity (CD206^+^).

*Glucose and LA uptake capacity assays for in vitro-generated T cells and Tregs:* Culture medium samples were collected at four specific time points: 12 hours, 24 hours, 36 hours, and 48 hours after the initiation of T-cell and Treg culture. For each sample collected at these time points, we strictly followed the protocol of the glucose detection kit to measure the glucose concentration in the medium. T cells and Tregs were cultured in medium supplemented with 10 mM lactate. The medium samples were collected at the same time points and analyzed via a lactate detection kit to measure the LA concentration.

*In vitro analysis of the effect of BMP4 on fibrosis*: Fibroblasts were spread in Petri dishes and incubated at 37 °C and 5% CO2 for 12 h. First, 1 mg/mL TGF-β was added to the control group, and 1 mg/mL TGF-β and 50 ng/mL BMP4 protein were added to the experimental group, which was then treated for 2 days. The samples were subsequently stained with DAPI, anti-TGF-β (1:1000 dilution) and anti-α-SMA (1:1000 dilution), and different channels, as well as merged channels, were captured with a Leica confocal microscope fluorescence image.

*Synthesis of PLGA nanoparticles*: The primary emulsion was prepared by dissolving 20 mg of PLGA in 2 mL of dichloromethane as the oil phase and adding 200 μL of LA as the aqueous phase to the oil phase, and the mixed solution was sonicated for 4 min. Afterwards, the primary emulsion was added to 10 mL of 1% PVA solution and sonicated for 2 min to prepare the compound emulsion. The compound emulsion was added to 10 mL of 0.33% PVA solution, evaporated at room temperature for 5 h in a window, washed three times with ultrafiltration centrifuge tubes at 1000 rpm for 5 min, vacuum lyophilized and stored at -20 °C.

*Fabrication of GelMA-PLGA@LA-BMP4 and PLA scaffolds*: The PLA pellets were loaded into a printing syringe, heated at 200 °C for 1 h until completely melted, and printed into a lattice-like PLA scaffold via an electrostatic spinning extruder printer at voltages of 4.7 kV and 1000 mm/min. PLGA nanoparticle suspensions, 50 ng/mL BMP4 and 0.5% photo crosslinker I2959 were mixed into GelMA at 1:1:2, and the mixed solution was frozen at 4 °C for 20 min until jelly like, printed *in situ* on PLA scaffolds via an extruded 3D printer at 100 mm/min, and irradiated with UV light for 1 min for crosslinking.

*Detection of the PLA output voltage:* A material testing system was employed to apply a series of precisely controlled quasistatic mechanical stimuli to the PLA scaffold samples, exerting forces of 0.01 N, 0.05 N, and 0.10 N, respectively. A high-impedance oscilloscope directly connected to the scaffold's two electrodes monitored and recorded the transient output voltage signals generated under these dynamic mechanical loads in real time.

*Preparation of PLGA@LA-BMP4-PG*: The PLGA nanoparticle suspension, 50 ng/mL BMP4, and 0.5% photocrosslinker I2959 were mixed with GelMA at a ratio of 1:1:2. The resulting mixture was then frozen at 4 °C for 20 minutes until it reached a jelly like consistency. It was subsequently printed into a mesh hydrogel via an extruded 3D printer at a speed of 100 mm/min, followed by UV irradiation for 1 minute to induce crosslinking. PLA pellets were loaded into a printing syringe and heated to 200 °C for one hour until fully melted. The molten PLA was then printed into a lattice-like scaffold via an electrostatic spinning extruder printer at a voltage of 4.7 kV and a speed of 1000 mm/min. Finally, the PLA scaffolds were mounted with the crosslinked hydrogel.

*Characterization of the PLGA@LA NP, GelMA-PLGA@LA-BMP4 and PLA scaffolds*: The size, surface charge and morphology of the PLGA@LA NPs were measured via DLS and TEM. The size and morphology of the GelMA-PLGA@LA-BMP4 and PLA scaffolds were measured via SEM. The piezoelectricity of the PLA mounts was evaluated via a TH2690 Electrometer/High Resistance meter.

All tests on mechanical properties were carried out in a universal mechanical testing machine, and tensile strength and adhesion were calculated as follows:

Tensile strength = maximum load/cross-sectional area.

Compression strength = maximum load/cross-sectional area.

Adhesion force = maximum load/adhesion area.

*Drug release from PLGA@LA-BMP4-PG:* The scaffold was immersed in pH 7.4 PBS buffer solution. The samples were placed in a constant-temperature shaking incubator at 37 °C for continuous agitation to simulate the *in vivo* environment. The scaffold samples were then collected at predetermined time points (days 1, 2, 3, 4, 5, 6, 7, 8, 9, and 10). The collected eluate was analyzed via an LA detection kit to obtain dynamic data on the concentration (in mM) of LA released during degradation.

*PLA-facilitated drug release testing:* Two scaffold materials, PLGA@LA-BMP4-PG and PLGA@LA-BMP4-G (without the PLA scaffold), were immersed in pH 7.4 PBS buffer solution and placed in a 37 °C constant-temperature shaking incubator for continuous agitation to simulate the *in vivo* environment. At ten predetermined time points from Day 1 to Day 10, parallel samples of each scaffold were retrieved, gently rinsed with deionized water, freeze-dried to a constant weight, and weighed via a precision electronic balance. The weight loss rate (%) during degradation was calculated for each group, enabling quantitative analysis of their degradation kinetics.

*Biological safety of the PLGA@LA-BMP4-PG degradation products:* The scaffold was immersed in pH 7.4 PBS buffer and continuously agitated in a 37 °C constant-temperature shaking incubator. Degradation extracts were collected at predetermined time points (day 3, day 7, and day 10), which were then used as culture media for fibroblast and T-cell cultures. Cell proliferation was assessed via the CCK-8 assay. Fibroblasts in the proliferative phase were labeled and imaged via an EdU staining kit. Flow cytometry was employed for phenotypic characterization and quantitative analysis of Tregs within the culture system. This comprehensive approach evaluated the effects of scaffold degradation products on the proliferation and differentiation of specific cell subsets.

*Cytotoxicity evaluation of the Dual scaffolds*: Keratinocyte viability was detected via the Cell Counting Kit-8 (CCK-8) method. Keratin-forming cells were spread in 96-well plates, divided into six groups of six wells each, and cultured at 37 °C and 5% CO2 for 12 h. The cells were treated as follows: (1) PBS; (2) PG (PLA-GelMA); (3) BMP4-PG; (4) PLGA@LA-PG; (5) PLGA@LA-BMP4-PG; PLA and GELMA, 5 mm*5 mm in size. The final concentration of LA was 10 mM, and the final concentration of BMP4 was 50 ng/mL. After 1 and 3 days of treatment, 100 µL of working medium (DMEM:CCK8=10:1) was added to the wells, the samples were incubated for 2 h in the dark, and the absorbance was detected at 450 nm via an enzyme marker.

*In vitro live‒dead cell staining*: Keratin-forming cells were inoculated in 24-well plates and incubated for 24 h at 37 °C and 5% CO2. The cells were treated for 72 h with (1) PBS, (2) PG, (3) BMP4-PG, (4) PLGA@LA-PG, or (5) PLGA@LA-BMP4-PG, and the sizes of the PLA and GelMA were 1 cm*1 cm. The final concentration of LA was 10 mM, and the final concentration of BMP4 was 50 ng/mL. Then, the corresponding wells were stained with calcein-AM/propidium iodide (PI) (50 μL, 20 mM) for 30 min, and the live and dead cells were observed via fluorescence microscopy.

*Cell migration*: Keratin-forming cells were spread in 24-well plates and incubated at 37 °C and 5% CO2 until they reached 100% confluence. A 200 µL lance tip was used to make a straight line mark in the wells, the medium in the well plates was replaced with serum-free basal medium, and the migration status of the cells was observed with a microscope and recorded at 0, 6, 12 and 24 h.

*For the tube formation assay*, two hundred microliters of matrix gel (Corning) was placed into a 24-well plate at low temperature and allowed to set for 1 h at 37 °C and 5% CO2. A total of 20,000 HUVECs were inoculated into each well, after which different groups of cells were treated. After treatment, the well plates were incubated at 37 °C and 5% CO2 for 3 h. After treatment, bright field images of tube formation were taken with a Leica microscope. ImageJ software (v1.53t) was used to quantify the number of nodes, extremities, segments, branches and meshes.

*Effect of T cells on the proliferation of HFSCs*: Hair follicle stem cells were seeded in 96-well plates and cultured overnight at 37 °C until they were seeded. After the plate was laid out, the cells were divided into 3 groups: (1) the Ctrl group; (2) the coculture with T cells; (3) the coculture with Treg cells, and the working medium DMEM:CCK8=10:1 was added to the well plates on days 1 and 3 after the treatments, and the absorbance at 450 nm was measured with an enzyme marker to assess the proliferation of hair follicle stem cells after different treatments.

*Proliferation of HFSCs*: HFSCs and T cells were seeded at a density of 50,000 cells per well in a 24-well plate and incubated at 37 °C with 5% CO₂ for 12 h. The cells were then subjected to the following treatments: (1) PBS; (2) PG; (3) BMP4-PG; (4) PLGA@LA-PG; and (5) PLGA@LA-BMP4-PG. A 2X EdU (purchased from Beyotime, C0075S) working solution (20 μM) was prepared, and equal volumes of the prewarmed solution were added to the wells. The cells were further incubated for 2 h to allow for EdU labeling, after which the culture medium was removed. The cells were subsequently fixed with 4% paraformaldehyde for 15 min. After fixation, the cells were washed and permeabilized, followed by the preparation of the click reaction solution and incubation at room temperature for 30 min. After washing, the cells were observed under a confocal laser scanning microscope (CLSM), where red fluorescence indicated proliferating cells.

*Proliferation of fibroblasts*: Fibroblasts were seeded at a density of 50000 cells per well in a 24-well plate and incubated at 37 °C with 5% CO₂ for 12 h. The cells were then subjected to the following treatments: (1) PBS; (2) PG; (3) BMP4-PG; (4) PLGA@LA-PG; and (5) PLGA@LA-BMP4-PG. A 2X EdU (purchased from Beyotime, C0075S) working solution (20 μM) was prepared, and equal volumes of the prewarmed solution were added to the wells. The cells were further incubated for 2 h to allow for EdU labeling, after which the culture medium was removed. The cells were subsequently fixed with 4% paraformaldehyde for 15 min. After fixation, the cells were washed and permeabilized, followed by the preparation of the click reaction solution and incubation at room temperature for 30 min. After washing, the cells were observed under a confocal laser scanning microscope (CLSM), where red fluorescence indicated proliferating cells.

*In vitro fibrosis analysis*: Fibroblasts were seeded onto confocal dishes and cultured at 37 °C with 5% CO₂ for 24 h. Subsequently, 1 µg/mL TGF-β was added to all the cells. The cells were then subjected to the following treatments: (1) PBS; (2) PG; (3) BMP4-PG; (4) PLGA@LA-PG; and (5) PLGA@LA-BMP4-PG. After 2 days of treatment, the cells were stained with DAPI, anti-TGF-β, and anti-α-SMA antibodies. Fluorescence images of different channels, as well as merged images, were captured via CLSM.

*In vitro flow cytometry analysis of T cells*: T cells were extracted from the spleens of mice at approximately 4 weeks of age, spread into 6-well plates and cultured for 1 day. The T cells were then subjected to the following treatments: (1) PBS; (2) PG; (3) BMP4-PG; (4) PLGA@LA-PG; and (5) PLGA@LA-BMP4-PG. After 3 days of treatment, the cells were collected and stained with anti-CD3 APC, anti-CD4 BV605, anti-CD8 FITC, anti-CD25 PE/Cy7 and anti-Foxp3 BV421 antibodies, followed by flow cytometry analysis of the proportion of each subpopulation.

*In vitro flow analysis of macrophages*: RAW 264.7 cells were treated with 1 µg/mL LPS for 24 h, and the macrophages were then subjected to the following treatments: (1) PBS; (2) PG; (3) BMP4-PG; (4) PLGA@LA-PG; and (5) PLGA@LA-BMP4-PG. After 2 days of treatment, the cells were collected and stained with anti-CD11b BV605, anti-CD86 BV650, and anti-CD206 PE/610 antibodies and then analyzed via flow cytometry to compare the M1 to M2 macrophage ratios of M1 to M2 macrophages.

*For in vitro IL-6 analysis*, RAW 264.7 cells were seeded on confocal dishes and incubated at 37 °C with 5% CO₂ for 6 h. Afterward, the cells were polarized with 1 µg/mL LPS for 12 h. The macrophages were then subjected to the following treatments: (1) PBS; (2) PG; (3) BMP4-PG; (4) PLGA@LA-PG; and (5) PLGA@LA-BMP4-PG. The cells were then stained with DAPI and anti-IL-6 (1:1000 dilution), and fluorescence images at different passages, as well as merged images, were captured via CLSM. RAW 264.7 cells were seeded on confocal dishes and incubated at 37 °C with 5% CO₂ for 6 h. Following polarization with 1 µg/mL LPS for 12 h, the macrophages were subjected to the following treatments: (1) PBS; (2) PG; (3) BMP4-PG; (4) PLGA@LA-PG; and (5) PLGA@LA-BMP4-PG for 12 h. The cell supernatant was collected for Western blotting analysis to determine the IL-6 secretion levels.

*In vitro analysis of the effects of IL-6 on fibrosis*: Fibroblasts were seeded in culture plates, and the Ctrl group was left untreated. In the experimental group, the cells were treated with 100 ng/mL IL-6 for 24 h after attachment. The cells were subsequently collected for Western blot analysis to measure the expression of the fibrosis marker α-SMA.

*Animal study*: BALB/c mice (female, 4–5 weeks, 20–25 g) were first anesthetized with isoflurane, the dorsal skin was sterilized, and a circular wound of 7 mm in diameter was formed with a perforator, after which different treatments were applied to the wound. The rabbits (New Zealand white rabbits, female, 3–4-month-old, 2–3 kg) were anesthetized with isoflurane, the abdominal tissues of the ears were disinfected, and a square incision with a side length of 1 cm was made with a scalpel, after which different treatments were applied to the incision.

*The effect of BMP4 on* *Treg polarization via macrophages:* A Transwell coculture system was established with M0 macrophages in the upper chamber and T cells in the lower chamber. The experimental group was stimulated with BMP4 in the upper chamber, whereas the Ctrl group received no treatment. After three days of coculture under standard conditions, T cells from the lower chamber were collected and stained with anti-CD4, anti-CD25, and anti-Foxp3 antibodies. The proportion of Tregs (CD4⁺CD25⁺Foxp3⁺) within the T-cell population was subsequently analyzed via flow cytometry.

*Statistics and Analysis of Transcriptomics:* In the volcano plot of transcriptomic data analysis, a 1.5-fold change (FC) threshold was applied for selecting upregulated and downregulated genes, with a statistical significance threshold set at P < 0.05. For GO enrichment analysis, the minimum gene set size was defined as 5, and the significance threshold was set at P < 0.01.

**Figure S1.** Quantitative analysis of TGF-β/a-SMA expression levels in each group (n=3). The data are presented as the means ± SDs. The P values were calculated via one-way analysis of variance (ANOVA). *P < 0.05, **P < 0.01, ***P < 0.001 and ****P < 0.0001.

**Figure S2.** Relative Quantitative Analysis of IL-4 by ELISA (n=3). The data are presented as the means ± SDs. The P values were calculated via one-way analysis of variance (ANOVA). *P < 0.05, **P < 0.01, ***P < 0.001 and ****P < 0.0001.

**Figure S3.** Relative Quantitative Analysis of IL-10 by ELISA (n=3). The data are presented as the means ± SDs. The P values were calculated via one-way analysis of variance (ANOVA). *P < 0.05, **P < 0.01, ***P < 0.001 and ****P < 0.0001.


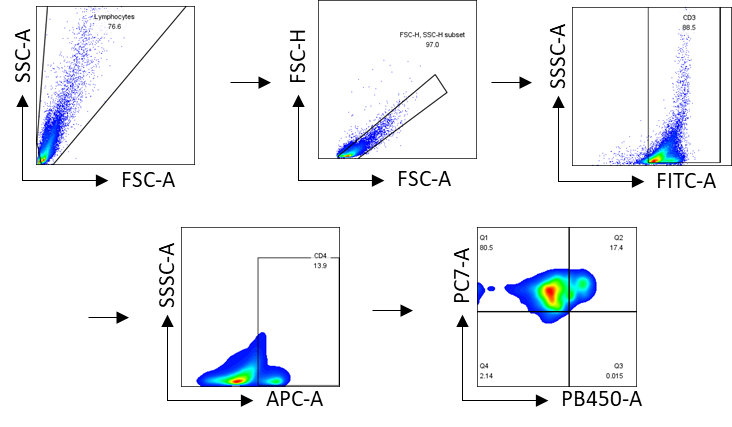


**Figure S4.** Gating strategy for FCM analysis of T cells.

**Figure S5.** Glucose concentration data at different time points (n=3). The data are presented as the means ± SDs. The P values were calculated via one-way analysis of variance (ANOVA). *P < 0.05, **P < 0.01, ***P < 0.001 and ****P < 0.0001.

**Figure S6.** LA concentration data at different time points(n=3). The data are presented as the means ± SDs. The P values were calculated via one-way analysis of variance (ANOVA). *P < 0.05, **P < 0.01, ***P < 0.001 and ****P < 0.0001.

**Figure S7.** Quantification of Tregs in CCK-8 assay results(n=3). The data are presented as the means ± SDs. The P values were calculated via one-way analysis of variance (ANOVA). *P < 0.05, **P < 0.01, ***P < 0.001 and ****P < 0.0001.


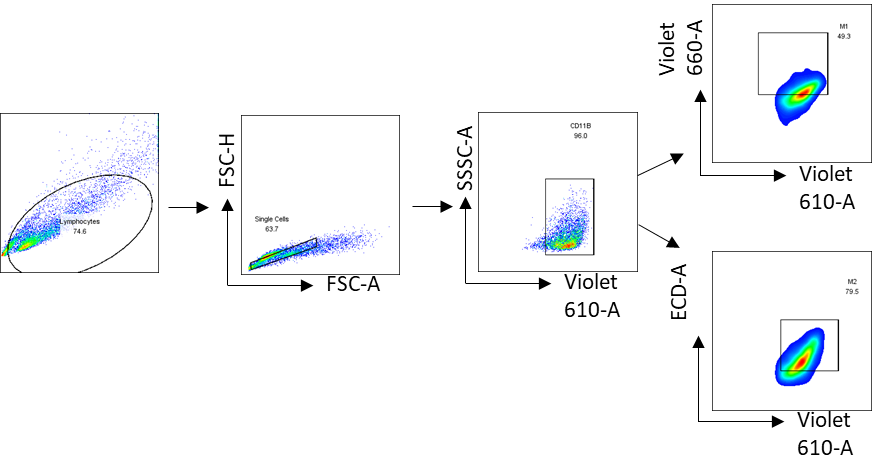


**Figure S8.** Gating strategy for FCM analysis of macrophages.


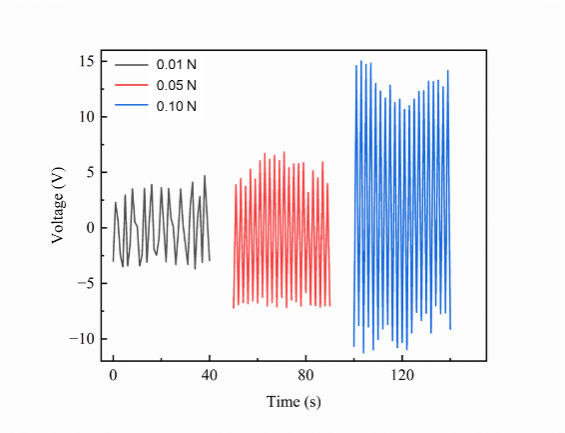


**Figure S9.** PLA voltage under different applied forces.

**Figure S10.** Particle size distribution of PLGA nanoparticles.

**Figure S11.** LA concentration.

**Figure S12.** PLA weight loss rate.


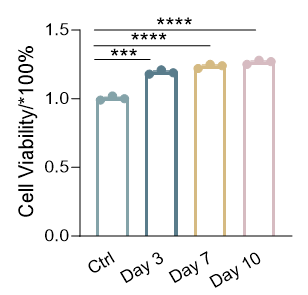


**Figure S13.** Cell viability at different time points of scaffold degradation (n=3). The data are presented as the means ± SDs. The P values were calculated via one-way analysis of variance (ANOVA). *P < 0.05, **P < 0.01, ***P < 0.001 and ****P < 0.0001.


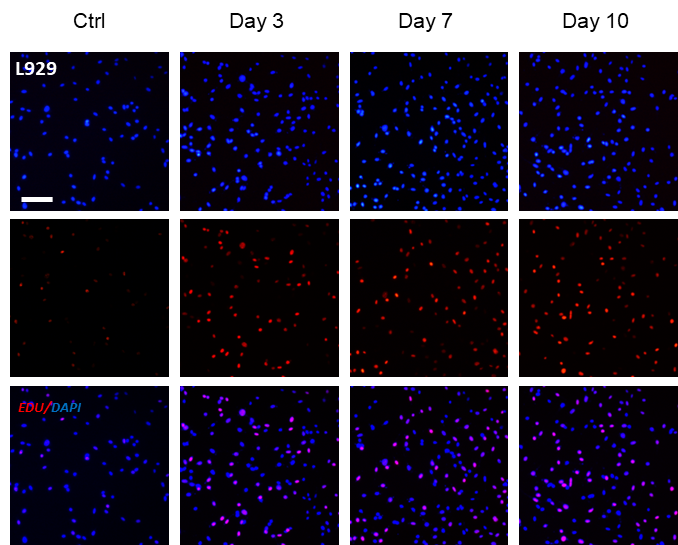


**Figure S14.** Fluorescence staining plots of EDU-labeled proliferating L929.

Scale bar: 100 μm.


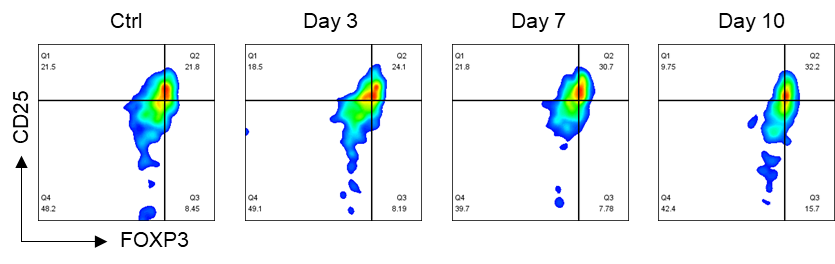


**Figure S15.** Flow cytometry analysis results of Tregs at different time points.


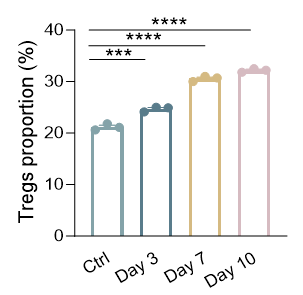


**Figure S16.** Quantitative results for flow cytometry analysis results of Tregs (n=3). The data are presented as the means ± SDs. The P values were calculated via one-way analysis of variance (ANOVA). *P < 0.05, **P < 0.01, ***P < 0.001 and ****P < 0.0001.

**Figure S17.** LA concentration.

**Figure S18.** GelMA weight loss rate.

**Figure S19.** CCK8 results of HFSCs proliferation on day 1 and 3 (n=3). The data are presented as the means ± SDs. The P values were calculated via one-way analysis of variance (ANOVA). *P < 0.05, **P < 0.01, ***P < 0.001 and ****P < 0.0001.


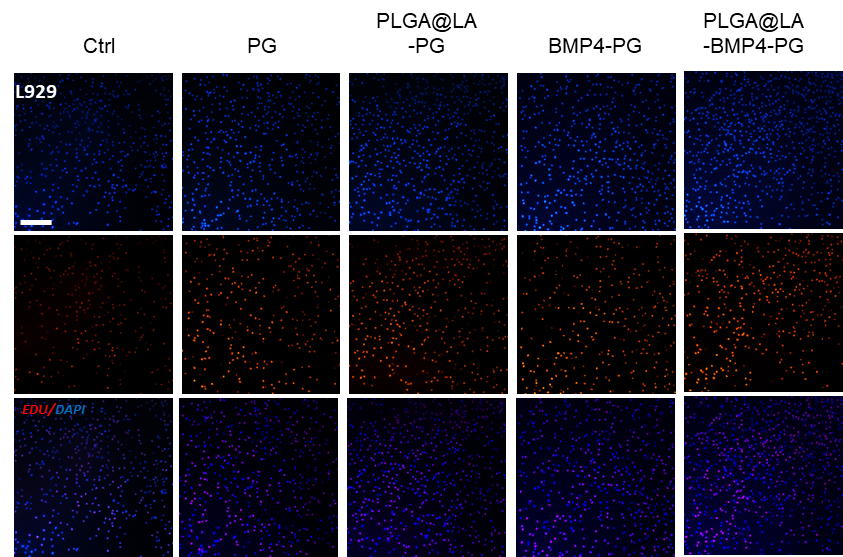


**Figure S20.** Fluorescence staining plots of EdU-labeled proliferating L929. Scale bar: 100 μm.

**Figure S21.** Quantification of EdU fluorescence intensity (n=3). The data are presented as the means ± SDs. The P values were calculated via one-way analysis of variance (ANOVA). *P < 0.05, **P < 0.01, ***P < 0.001 and ****P < 0.0001.


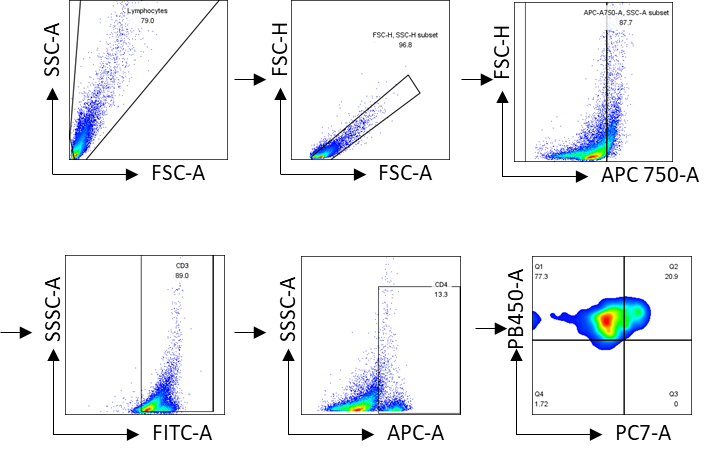


**Figure S22.** Gating strategy for FCM analysis of T cells.


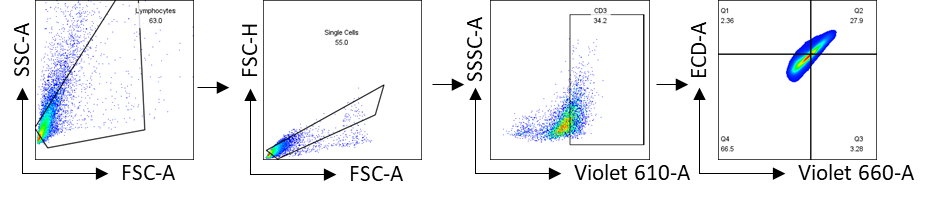


**Figure S23.** Gating strategy for FCM analysis of macrophages.


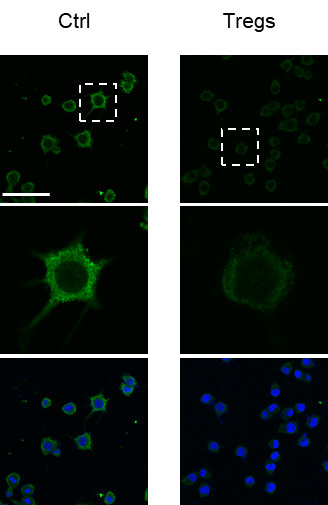


**Figure S24.** Representative fluorescence images of IL-6 expression in macrophages. Scale bars: 50 μm.

**Figure S25.** Quantification of fluorescent intensity of IL-6 expression in macrophages (n=3). The data are presented as the means ± SDs. The P values were calculated via one-way analysis of variance (ANOVA). *P < 0.05, **P < 0.01, ***P < 0.001 and ****P < 0.0001.

**Figure S26.** Quantification of fluorescent intensity of IL-6 expression in macrophages (n=3). The data are presented as the means ± SDs. The P values were calculated via one-way analysis of variance (ANOVA). *P < 0.05, **P < 0.01, ***P < 0.001 and ****P < 0.0001.

**Figure S27.** Quantification of western blot of IL-6 in macrophages (n=3). The data are presented as the means ± SDs. The P values were calculated via one-way analysis of variance (ANOVA). *P < 0.05, **P < 0.01, ***P < 0.001 and ****P < 0.0001.

**Figure S28.** Quantification of western blot of α-SMA in fibroblasts (n=3). The data are presented as the means ± SDs. The P values were calculated via one-way analysis of variance (ANOVA). *P < 0.05, **P < 0.01, ***P < 0.001 and ****P < 0.0001.


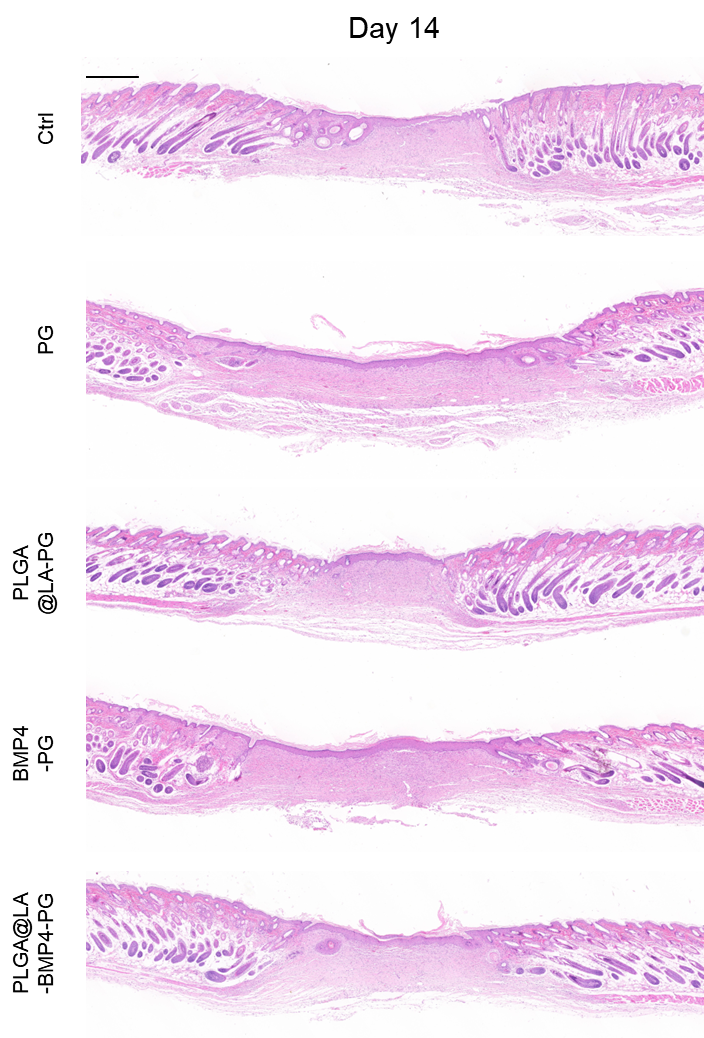


**Figure S29.** Representative images of H&E staining of wound tissue on day 14. Scale bar: 1 mm.

**Figure S30.** Quantification of the collagen volume fraction shown in Fig. 7a (n=3). The data are presented as the means ± SDs. The P values were calculated via one-way analysis of variance (ANOVA). *P < 0.05, **P < 0.01, ***P < 0.001 and ****P < 0.0001.

**Figure S31.** Quantification of Ⅰ/Ⅲ collagen in Fig. 7b on day 14 (n=3). The data are presented as the means ± SDs. The P values were calculated via one-way analysis of variance (ANOVA). *P < 0.05, **P < 0.01, ***P < 0.001 and ****P < 0.0001.

**Figure S32.** Quantification of Ⅰ/Ⅲ collagen in Fig. 7b on day 21 (n=3). The data are presented as the means ± SDs. The P values were calculated via one-way analysis of variance (ANOVA). *P < 0.05, **P < 0.01, ***P < 0.001 and ****P < 0.0001.

**Figure S33.** Quantification of fluorescent intensity of TGF-β expression on day 14 (n=3). The data are presented as the means ± SDs. The P values were calculated via one-way analysis of variance (ANOVA). *P < 0.05, **P < 0.01, ***P < 0.001 and ****P < 0.0001.

**Figure S34.** Quantification of fluorescent intensity of KRT17 expression on day 14 (n=3). The data are presented as the means ± SDs. The P values were calculated via one-way analysis of variance (ANOVA). *P < 0.05, **P < 0.01, ***P < 0.001 and ****P < 0.0001.

**Figure S35.** Quantification of fluorescent intensity of TGF-β expression on day 21 (n=3). The data are presented as the means ± SDs. The P values were calculated via one-way analysis of variance (ANOVA). *P < 0.05, **P < 0.01, ***P < 0.001 and ****P < 0.0001.

**Figure S36.** Quantification of fluorescent intensity of KRT17 expression on day 21 (n=3). The data are presented as the means ± SDs. The P values were calculated via one-way analysis of variance (ANOVA). *P < 0.05, **P < 0.01, ***P < 0.001 and ****P < 0.0001.


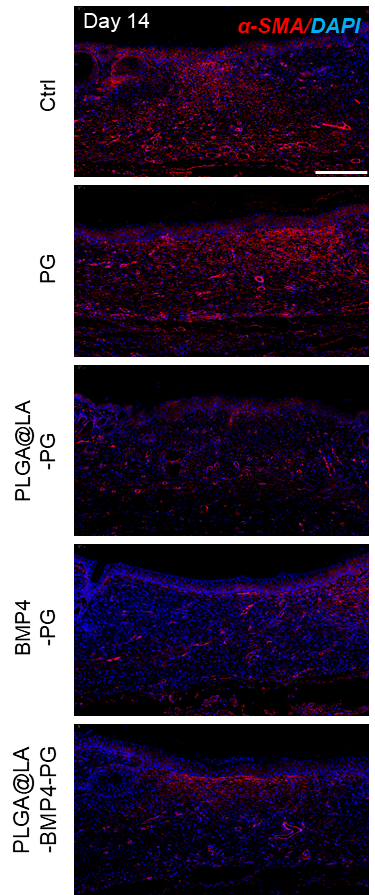


**Figure S37.** Representative images of α-SMA fluorescence staining of skin tissue on day 14. Scale bar: 200 μm.


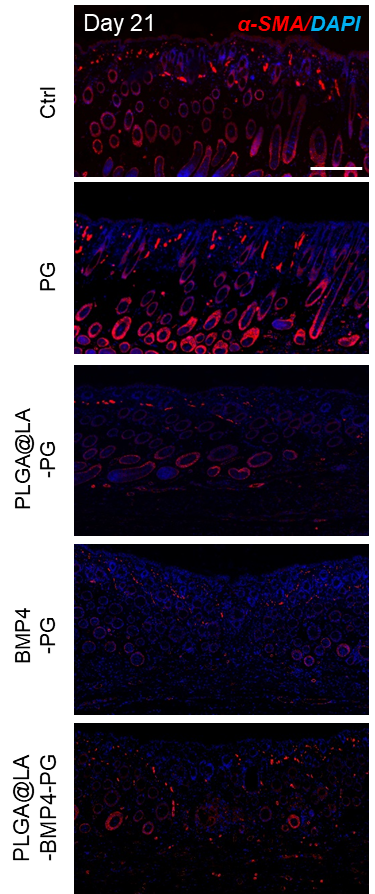


**Figure S38.** Representative images of α-SMA fluorescence staining of skin tissue on day 21. Scale bar: 400 μm.

**Figure S39.** Quantification of fluorescent intensity of α-SMA expression on day 14 (n=3). The data are presented as the means ± SDs. The P values were calculated via one-way analysis of variance (ANOVA). *P < 0.05, **P < 0.01, ***P < 0.001 and ****P < 0.0001.

**Figure S40.** Quantification of fluorescent intensity of α-SMA expression on day 21 (n=3). The data are presented as the means ± SDs. The P values were calculated via one-way analysis of variance (ANOVA). *P < 0.05, **P < 0.01, ***P < 0.001 and ****P < 0.0001.


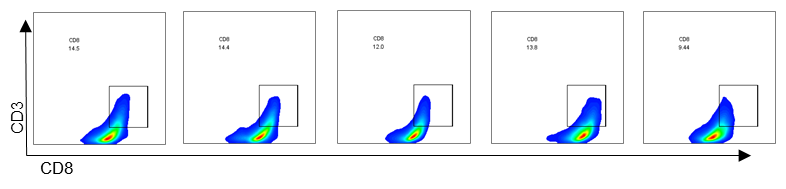


**Figure S41.** Representative plots of the results of flow cytometry analysis of CD8^+^ T-cell polarization.

**Figure S42.** Quantification of CD8^+^ T cells flow analysis results (n=3). The data are presented as the means ± SDs. The P values were calculated via one-way analysis of variance (ANOVA). *P < 0.05, **P < 0.01, ***P < 0.001 and ****P < 0.0001.


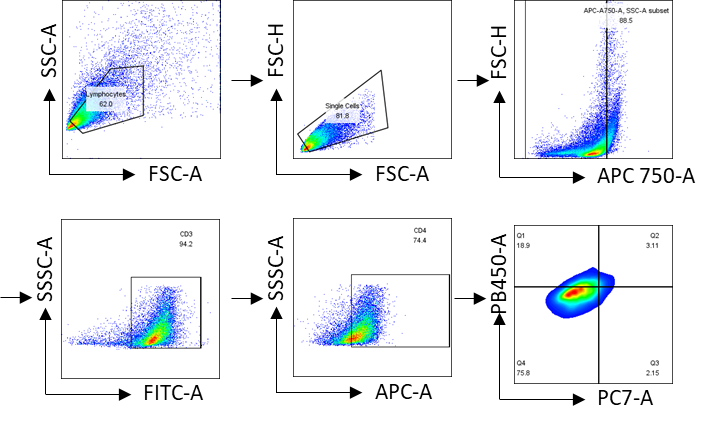


**Figure S43.** Gating strategy for FCM analysis of T cells.


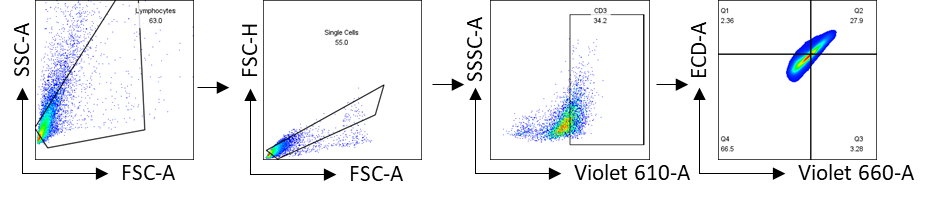


**Figure S44.** Gating strategy for FCM analysis of macrophages.


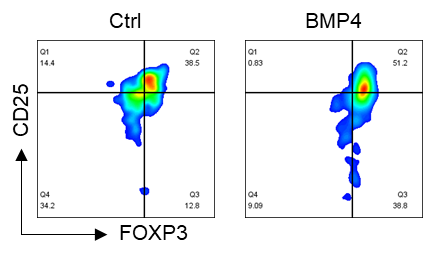


**Figure S45.** Flow cytometry analysis results for Tregs.

**Figure S46.** Quantitative results for flow cytometry analysis results of Tregs (n=3). The data are presented as the means ± SDs. The P values were calculated via one-way analysis of variance (ANOVA). *P < 0.05, **P < 0.01, ***P < 0.001 and ****P < 0.0001.


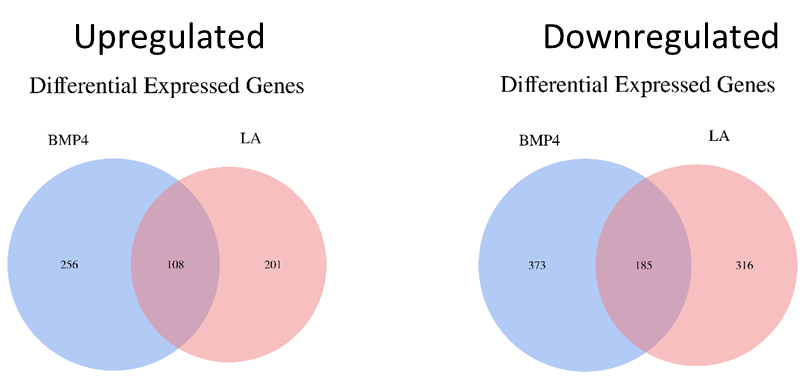


**Figure S47.** Venn diagram of upregulated and downregulated DEGs.


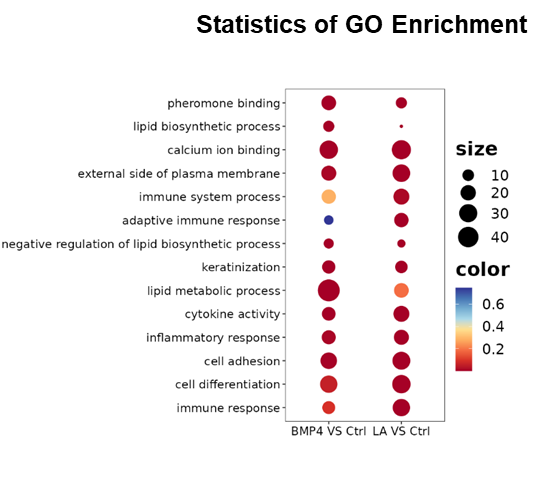


**Figure S48.** Statistics of GO enrichment.


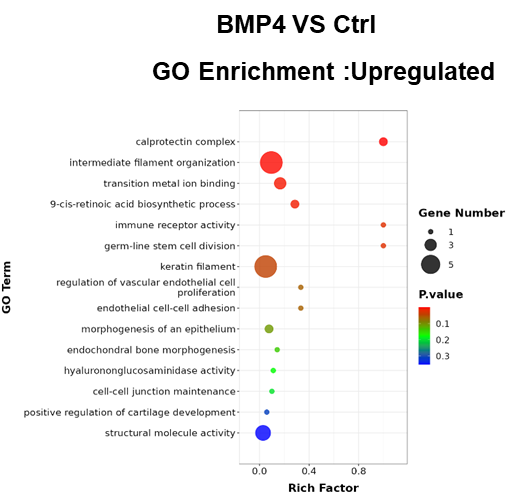


**Figure S49.** Upregulated enriched GO terms in the BMP4 VS Ctrl group.


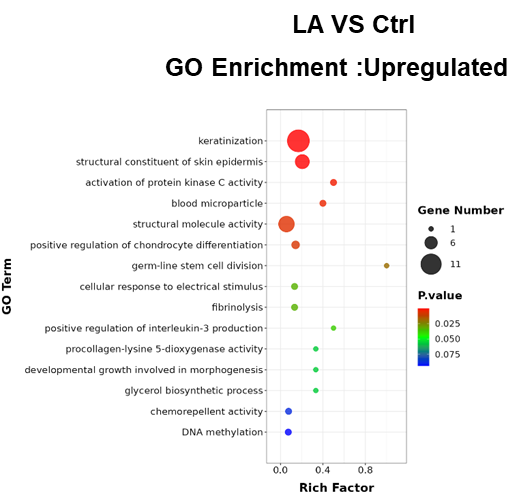


**Figure S50.** Upregulated enriched GO terms in the LA vs Ctrl group.
